# How much habituation is enough? An R application for ad hoc content-related limits of acceptance in behavioral research

**DOI:** 10.3758/s13428-026-03170-2

**Published:** 2026-09-30

**Authors:** Konstantin Warneke, Stanislav D. Siegel, José Afonso, Marco Herbsleb, Sebastian Wallot

**Affiliations:** 1 Institute of Psychology, Leuphana University Lüneburg, Lüneburg, Germany; 2 Department of Human Movement Science and Exercise Physiology, Friedrich Schiller University Jena, Jena, Germany; 3 Centre of Research, Education, Innovation, and Intervention in Sport (CIFI2D), Faculty of Sport, University of Porto, Porto, Portugal; 4 Department of Sports Medicine and Health Promotion, Friedrich Schiller University Jena, Jena, Germany; 5 Department of Psychosomatic Medicine and Psychotherapy, University Hospital Jena, Jena, Germany

**Keywords:** Limits of agreement, Repeatability, R-Script, Reliability, Neurocognitive accuracy, Practice effects

## Abstract

Evaluating test–retest reliability is of crucial importance for clinical decision-making and an integral component of studies in empirical sciences. In psychology, classical test theory focused on reliability coefficients, but research has shown that intraclass correlation coefficients and Cronbach’s alpha have limited value in quantifying individual measurement error sources appropriately. Preliminary assumptions such as parallel readings and independency of error sources are violated by practice/habituation effects, i.e., test–retest systematic errors. Although awareness of the need for additional measurement quantifications to properly assess test–retest agreement has increased, many such quantifications are insufficiently implemented. Furthermore, one of the most important agreement analyses introduced by Bland and Altman in 1986 has interpretation limitations, as the limits of agreement are descriptive and not linked to content-based clinical decision-making, oftentimes causing inappropriate usage. To counteract these limitations and improve the implementation of agreement analyses in addition to more commonly used reliability coefficients, we introduce a software package that allows predefining acceptable measurement limits (limits of acceptance) to evaluate the sufficiency of task habituation to gauge individual error sources. The package implements a comprehensive test–retest evaluation in order to improve transparency of data quality reporting in psychological and cognitive research.

**Supplementary Information:**

The online version contains supplementary material available at https://doi.org/10.3758/s13428-026-03170-2.

## Introduction

In empirical sciences such as psychology or neurocognitive research, reliability is a necessary, but insufficient, precondition for establishing internal and external validity of results (Patino & Ferreira, 2018). Broadly, reliability refers to the consistency of a measurement tool, procedure, or device in capturing the same characteristic under (mostly) identical conditions (Barnhart et al., 2007; Bartlett & Frost, 2008). Lachin (2004) defines it as the extent to which a measurement is reproducible and free from errors. Originally, reliability was defined as the ratio of the true score variance to the observed total score variance (Cronbach et al., 1972; Lord & Novick, 1986). It was developed for assessing the within-observer error in repeated measures under identical conditions (i.e., test–retest reliability), or to identify the discrepancy between two readings from two different instruments (i.e., validity, assessing how an instrument compares to a gold standard) or between observers on the same subject (i.e., objectivity or inter-rater reliability) (Barnhart et al., 2007). Appropriate quantification of reliability is decisive in empirical research, where measurement error is inherent.

In psychological and cognitive testing, a prominent approach for reliability evaluation adopts classical test theory (CTT), assuming X=T+E, with *X* being the observed score, resulting from the sum of a true score *T* and random error *E* (Medvedeva et al., 2023; Miller, 2009). Herein, the reliability coefficient is the ratio of the “true score” variance (${\sigma}_{T}^{2}$) to the “observed score” variance (${\sigma}_{X}^{2}$). Four assumptions should be fulfilled: 1) *T* and *E* are additive; 2) *T* and *E* are independent; 3) *E* is random; and 4) in repeated measures, *T* does not change (Collins, 2007). Krabbe (2017) described that readings must be parallel, meaning that each assessed item (e.g., question, statement) was valid to evaluate the intended construct and no significant test–retest error occurred. Statistically, this means that there is no significant difference between the test–retest data and that the only occurring measurement error is random (Barnhart et al., 2007; Bartlett & Frost, 2008; Krabbe, 2017).

By definition, the calculation of the reliability coefficient assumed the presence of a stable “true” score value (Barnhart et al., 2007; Collins, 2007, p. 9). In practice, these assumptions are oftentimes contradicted by practice/test habituation effects, causing a significant and systematic test–retest performance improvement (Del Rossi et al., 2014; Donath et al., 2017; Lo et al., 2012). Additionally, the assumption of the error variance being an independent and single factor (Medvedeva et al., 2023) was questioned by the Generalizability theory (GT), an extension of the CTT that splits the error variance into several sources and accounts for multiple origins (Barnhart et al., 2007; Cronbach et al., 1972; Medvedeva et al., 2023). Common correlation-based reliability coefficients like Cronbach’s α (Collins, 2007) or intraclass correlation coefficients (ICC) (Bogdanov et al., 2025; Koo & Li, 2016) limit interpretability of the complete construct of test–retest reliability, as the pure reviewing of these coefficients prohibits a detailed perspective of different relevant measurement error sources (Kottner & Streiner, 2011; Warneke et al., 2025a).

These limitations can be counteracted by the implementation of measurement error analyses as a supplement to reliability coefficients, accounting for different error sources that are commonly conflated in typical test–retest reliability assessments (Atkinson & Nevill, 1998; Bogdanov et al., 2025; Hopkins, 2000; Warneke et al., 2025d). In the following, we will briefly summarize different sources of measurement errors occurring in cognitive and behavioral research to underline the relevance of test–retest reliability with respect to internal data validity. Afterward, we will dive into the statistical quantification approaches and propose solutions to some of their limitations.

## Definitions and terms in test–retest reliability research

Since reliability is a general term and construct, oftentimes used in numerous settings to describe specific constructs, we will briefly revisit definitions of terms and constructs that will be used in this work, while calculation formulas will be provided later in this work to avoid redundancies.

Reliability is originally defined as an estimate of the true measurement construct that accounts for variability and error across multiple independent readings (Brennan, 2010; Cronbach, 1947). In its most intuitive sense, reliability simply means that a new measurement or retest value is the same as the truth or agrees with the truth (Barnhart et al., 2007). This is in accordance with the abovementioned test–retest variance splitting in the true score variance and the random error variance in CTT. However, a true score is not always measurable in test–retest evaluations. In contrast, in test–retest reliability analyses, as well as in interrater reliability assessment, the true score is hardly accessible, and oftentimes readings are not parallel, as systematic test–retest discrepancies are present. In this work, we will focus on test–retest reliability, although numerous aspects might fit other reliability constructs.

**Fig. 1:**
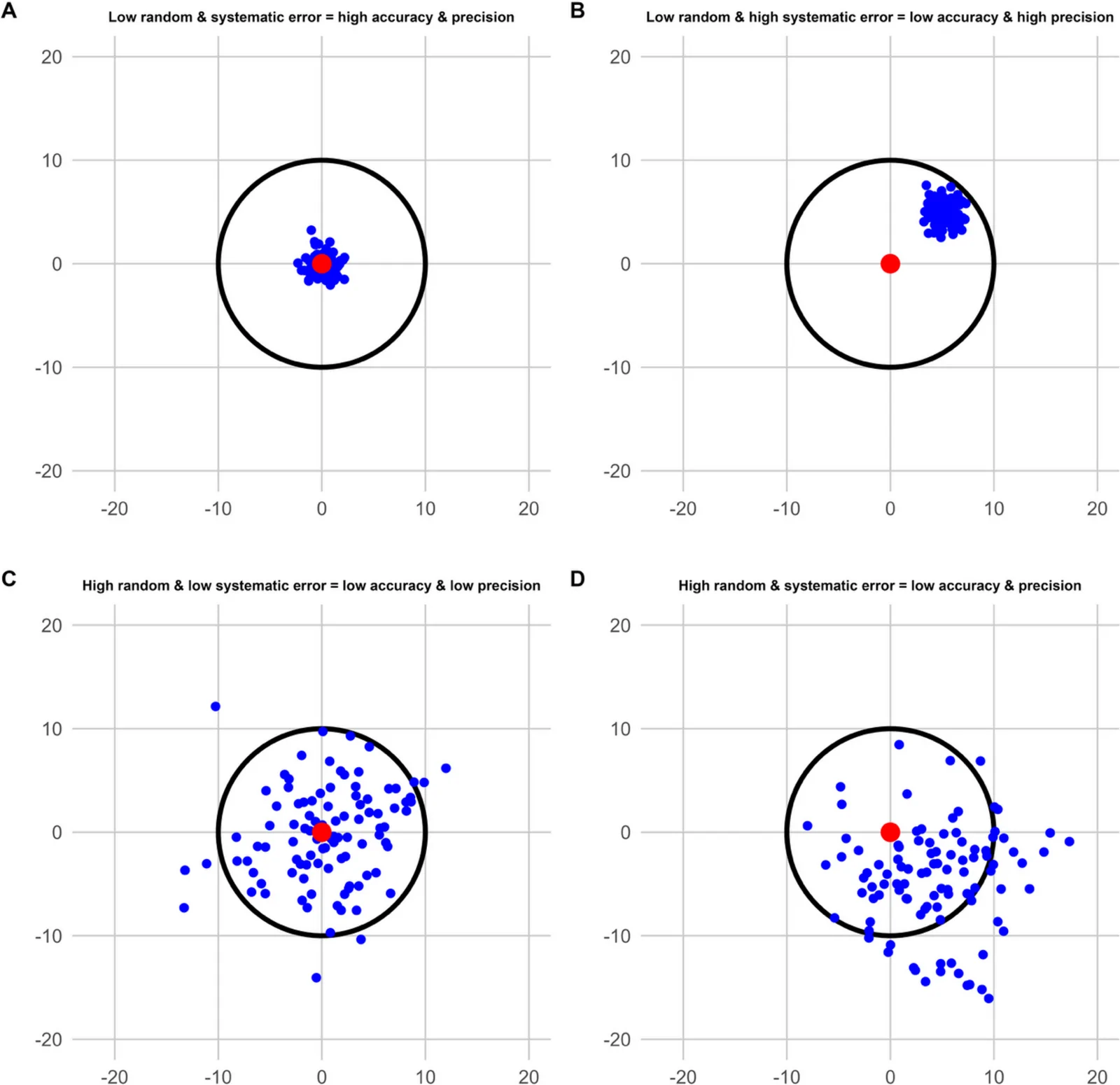
Illustration of the concepts of precision and accuracy using target shooting example by **A** (high precision, high accuracy): showing accurate testing (low random and no systematic error), **B** (high precision, low accuracy): inaccurate testing by precisely hitting the wrong spot, **C** (low precision, but correct value): inaccurate measurement by imprecisely hitting the right spot and **D** (low precision, without testing for the correct value): inaccurate testing by imprecisely hitting the wrong spot

A term oftentimes used as a synonym of reliability is repeatability, which seeks to quantify the error occurring when repeating the measurement directly in a row, without changing any circumstances or “the variation in repeated measurements made on the same subject under identical conditions” (Bartlett & Frost, 2008, p. 467). When performed directly in a row, it is considered the purest form of assessing the closeness of agreement between the test and the retest (Barnhart et al., 2007; Food and Drug Administration (FDA), 2001; International Organization of Standardization, 2023). This describes a specific case of measurement precision. More broadly, precision could be defined as the closeness of agreement under stipulated or identical conditions (Barnhart et al., 2007). However, precision can be large despite readings deviating from the true value. This differs from accuracy, which refers to precisely hitting the right spot and depends on the existence of a gold standard against which the test can be compared. Both constructs are graphically illustrated in Fig. 1 using target shooting to underline the difference.

Because no generally accepted definition of reliability exists, Table 1 summarizes how the term is used and the corresponding calculation formulas synthesized from the included literature.

**Table 1:** summarizes the used definitions and terms used in the following, which were synthesized from the literature

Term	Definition	Example for illustration	Common quantifications
Reliability	Reliability as the combination of repeatability and reproducibility (Hopkins, 2000) Reliability can be defined as the consistency of measurements or of an individual’s performance, on a test or “the absence of measurement error (Atkinson & Nevill, 1998) Reliability is the ratio of true score variance to the observed total score variance (CTT) Cronbach et al., 1972, Lord & Novick, 1986) to detect the measurement error if an observer takes a measurement repeatedly on the same subject (test–retest reliability) under identical conditions or the consistency of two readings collected by two different instruments on the same subject under identical conditions (device objectivity) (Barnhart et al., 2007, p. 535) A reliable test can estimate the true scores between individuals over time and minimizes error within the measurement (Medvedeva et al., 2023) Reliability is defined as the extent to which measurements can be replicated. It reflects correlation and agreement between measurements (Koo & Li, 2016, p 155,156). Reliability relates the magnitude of measurement errors in observed measurements to the inherent variability of error free, true level of quantity between subjects (Bartlett & Frost, 2008, p 467).	A shooter’s arrows consistently land in the same region of the target across multiple attempts, demonstrating stable performance	Correlation coefficients, intraclass correlation coefficients, Cronbach’s α
Intraday/intrasession reliability	Reliability within a day but between different readings per testing session	A shooter fires two shots back-to-back under identical conditions and achieves nearly the same outcome	Correlation coefficients, intraclass correlation coefficients, Cronbach’s alpha α
Intraday reliability	Reliability between days	A shooter practices on two different days and obtains similar scores or grouping patterns, despite the passage of time	Correlation coefficients, intraclass correlation coefficients, Cronbach’s alpha α
Repeatability	The degree to which test results remain stable under identical conditions within a single session (Barnhart et al., 2007) The closeness of agreement between independent test results under repeatability conditions that are as constant as possible (International Standardization Organization, in Barnhart et al., 2007) Measurement repeatability is the variation in repeated measurements made on the same subject under identical conditions (Barlett & Frost, 2008)	A shooter repeatedly hits the same spot on the target within one session, showing minimal variation in consecutive shots	Agreement analyses, Lin’s concordance correlation coefficient
Reproducibility	Reproducibility is the precision between different laboratories (Food and Drug Administration, 2001). Reproducibility is the closeness of agreement between independent test results under reproducibility conditions with the same method on the same test items, but in different laboratories and different operators and using different equipment (International Organization for Standardization, in Barnhart et al., 2007) Reproducibility is the variation in measurements made on the subject under changing conditions (Bartlett & Frost, 2008)	A shooter uses a different bow but still achieves similar performance outcomes, indicating the method’s robustness	Agreement analyses, Lin’s concordance correlation coefficient
Agreement	Agreement quantifies how close two measurements on the same subject were when measured on the same scale (Bartlett & Frost, 2008) Agreement is the closeness between readings and includes both accuracy and precision (Barnhart et al., 2008)	A shooter repeatedly hits the same spot on the target within one session and between different sessions, showing minimal variation in consecutive shots and hitting the target	Agreement analysis, Lin’s Concordance correlation coefficient
Precision	The closeness of agreement or the degree of scatter between a series of measurements collected from multiple sampling under prescribed conditions (Food and Drug Administration 2001) Precision is the closeness of agreement between independent test results obtained under stipulated conditions (International Organization for Standardization, 2023) in Barnhart et al., 2007 Reliability was equalized with precision: “It offers a definition of measurement precision known as reliability” (Collins, 2007, p.440)	A shooter’s arrows form a tight cluster, even if that cluster is not necessarily on the bullseye	Correlation coefficients, intraclass correlation coefficients, Cronbach’s α, Agreement analyses, Lin’s concordance correlation coefficient
Accuracy	The degree to which measurements match the true or intended value, considering systematic mean differences as well as the random error (Barnhart et al., 2007)	A shooter’s arrows are tightly grouped and also land on the bullseye, reflecting both precision and correctness of aim	Correlation coefficients, Intraclass correlation coefficients, Cronbach’s α, Agreement analyses, Lin’s concordance correlation coefficient, paired-sample *t* test, analysis of variance
Random error	Unpredictable fluctuations in measurements caused by chance factors, majorly stemming from biological sources or standardization problems (Hopkins, 2000, Atkinson & Nevill, 1998)	The shooter’s arrows land in different spots each time due to uncontrollable elements like sudden muscle twitches or brief gusts of wind	Agreement analyses, especially limits of agreement in Bland–Altman analyses, Hopkins’ typical error, standard error of measurement
Systematic error	A consistent, directional bias in measurements, often due to calibration issues or a flawed test setup as well as habituation/learning effects (Warneke et al., 2025a; Atkinson & Nevill, 1998)	A misaligned bow sight causes every arrow to veer off to the left by the same margin, no matter how many shots are taken	Agreement analyses, paired-sample *t* test, analysis of variance

Statistically, precision mainly relates to the magnitude of random error, oftentimes stemming from standardization limitations and/or biological variability (Atkinson & Nevill, 1998; Warneke et al., 2025a). When reaching the level of significance or reflecting a clinically relevant discrepancy, the systematic error compromises the assumption of parallel readings (Krabbe, 2017; Miller, 2009) and contradicts the calculation of reliability coefficients that assume no systematic discrepancy (Bartlett & Frost, 2008). A typical root cause of systematic errors in psychological or cognitive sciences is practice effects that affect test–retest reliability (Donath et al., 2017).

## Practice effects and systematic bias

Test–retest improvements in coordinative, cognitive, or psychological readings without performing any intervention between the test occasions are known as practice- or habituation effects (Schworer et al., 2023; Warneke et al., 2025d; White et al., 2019), usually occurring when participants are confronted with unfamiliar testing conditions (Collie et al., 2003; Estevis et al., 2012). Habituation effects (i.e., test–retest improvements within days or weeks in the absence of any intervention) are usually more pronounced between the first test–retest comparisons. Short-term, uninstructed test-to-test performance improvements over days or even weeks have been reported for a wide range of cognitive and executive function measures, including visuomotor tasks, interference inhibition tasks, and selective attention tests such as the Simon task, Eriksen flanker task, Stroop test, Wechsler Memory Scale, Digit Symbol Substitution Test, and Rapid Screen of Concussion. These studies show the largest gains between the first testing sessions, followed by a progressively flatter curve as participants become more familiar with the tasks, until no significant mean bias is observed between later testing days (Eileen De Monte et al., 2005; Theisen et al., 1998; Warneke et al., 2025d; White et al., 2019).

Similarly, long-term task learning effects from previous task performances up to years ago affected test scores in tests that were performed partly years after (e.g. the Halstead-Reitan Neuropsychological Test battery (Dikmen et al., 1999), the Wechsler Adult Intelligence Scale Fourth Edition (WAIS-IV) (Estevis et al., 2012) or the Wechsler Memory Scale III (Lo et al., 2012), showing the relevance of assessing specific task experience when testing for cognitive function (Collie et al., 2003; Estevis et al., 2012). These findings underline the relevance of sufficient familiarization with unhabituated tasks to avoid confusion between habituation effects and genuine changes, which might meaningfully bias clinical derivations (Bennett et al., 2017; Hopkins, 2000; Theisen et al., 1998).

## Random errors and biological variance

As acknowledged in CTT and GT, random errors play a significant role when quantifying reliability (e.g., test–retest reliability). Quantifying and minimizing measurement error is relevant to internal (data) consistency and validity. Random errors play an important role as they might stem from various sources that can be either controlled via test standardization (e.g., tests performed always at the same time of the day, with the same investigator, homogeneous sample with comparable task experience, etc.) or remain inherent (e.g., biological variability, changes in day-to-day situational influences) (Hopkins, 2000). Although measurement error cannot be avoided entirely, its appropriate quantification remains inconsistent.

The quantification of the random error deserves enhanced attention, excellent reliability (e.g., ICC ≥ 0.9) (Koo & Li, 2016) can be accompanied by meaningful measurement errors, with a test–retest mean and maximum absolute test–retest error of 20% and 90%, respectively (Atkinson & Nevill, 1998; Lamb, 1998; Warneke et al., 2025a). Accordingly, a detailed measurement error analysis (how close is the agreement between two measurements within a subject on the same scale) to improve interpretability and to better account for the different sources of measurement errors should supplement reliability coefficient reporting (Bogdanov et al., 2025; Galpin et al., 2008; Lempke et al., 2023; Lynall et al., 2021; Sanders et al., 2018; Wilke et al., 2020; Wilke & Vogel, 2020). In the following, the most common quantification approaches are synthesized from the literature.

## Test–retest reliability quantifications: Calculation of common metrics

Before providing formulas for reliability metrics, we present a definition of some of the basic terms. Let ${X}_{it}$ denote the observed score of the individual i at measurement time t, with i=1,⋯,n and t=1,⋯,k. In the present application, the focus is restricted to two repeated measurements; therefore, k=2, where ${X}_{i1}$ represents the first measurement and ${X}_{i2}$ represents the second measurement for individual i.

For each measurement occasion, the arithmetic mean is calculated as

1 $${\overline{X}}_{\cdot t}=\frac{1}{n}\sum_{i=1}^{n}{X}_{it}$$

Accordingly, the means of the first and second measurement times are obtained by setting t=1 and t=2, respectively. The corresponding standard deviation for each measurement occasion is

2 $$S{D}_{t}=\sqrt{\frac{{\sum_{i=1}^{n}({X}_{it}-{\overline{X} }_{\cdot t})}^{2}}{n-1}}$$

Because the following discussion focuses on test–retest quantifications with two repeated measurements, a pooled standard deviation is calculated as

3 $$S{D}_{\mathrm{p}\mathrm{o}\mathrm{o}\mathrm{l}\mathrm{e}\mathrm{d}}=\sqrt{\frac{S{D}_{1}^{2}+S{D}_{2}^{2}}{2}}$$

This pooled standard deviation has the advantage that it does not depend on which of the two repeated measurements is considered the test and which one was the retest. For the quantification of systematic bias and agreement, individual paired differences and participant-wise means are calculated. The paired difference for participant i is

4 $${d}_{i}={X}_{i2}-{X}_{i1}$$

whereas the participant-wise mean is

5 $${m}_{i}=\frac{{X}_{i1}+{X}_{i2}}{2}$$

### Calculation of intraclass correlation coefficients (ICC)

The underlying mathematics of the ICC reflects an analysis of variance (ANOVA) approach, which is expressed as a correlation coefficient. For the calculation of the ICC, the sum of squares of all included values (sum of squares total) is divided into between- and within-subject sum of squares (i.e., the repeated measurements). The exact calculation depends on the type of ICC, and the formulas will be presented below.

There are six ICC variants commonly used in the literature: ICC(1,1), ICC(1,2), ICC(2,1), ICC(2,2), ICC(3,1), and ICC(3,2), that were presented by Shrout & Fleiss (Shrout & Fleiss, 1979) and subsequently summarized by Koo & Li (2016) to the currently most-cited guidelines on how to choose the correct ICC. The choice for the correct ICC variant depends on three questions (Shrout & Fleiss, 1979): i) Is a one- or a two-way ANOVA preferable? ii) is there an important difference between judges’ mean ratings? iii) is the rating unit an individual rating or a mean of multiple ratings?

In the present application, k=2; the notation k (i.e., number of repeated measurements per observation unit) is retained to make the distinction between single-measure and average-measure ICCs explicit. To derive the ICCs, participant means, measurement occasion means, and the grand mean are calculated first. The measurement occasion mean is defined in Eq. 1. The participant-wise mean is calculated as

6 $${\overline{X}}_{i\cdot }=\frac{1}{k}\sum_{t=1}^{k}{X}_{it}$$

For the present case with two repeated measurements, this corresponds to Eq. 5. The grand mean across all observations is

7 $${\overline{X} }_{\cdot \cdot }=\frac{1}{n*k}\sum_{i=1}^{n}\sum_{t=1}^{k}{X}_{it}$$

For ICC(1,1) and ICC(1,k), the application uses the one-way random-effects formulation. The between-individual sum of squares is

8 $$S{S}_{\mathrm{i}\mathrm{n}\mathrm{d}\mathrm{i}\mathrm{v}\mathrm{i}\mathrm{d}\mathrm{u}\mathrm{a}\mathrm{l}}=k\sum_{i=1}^{n}{\left({\overline{X}}_{i\cdot }-{\overline{X} }_{\cdot \cdot }\right)}^{2}$$

the within-individual for the different measurement time points (here: k=2) sum of squares is

9 $$S{S}_{\mathrm{t}\mathrm{i}\mathrm{m}\mathrm{e}}=\sum_{i=1}^{n}\sum_{t=1}^{k}{\left({X}_{it}-{\overline{X}}_{i\cdot }\right)}^{2}$$

and the total sum of squares is

10 $$S{S}_{\mathrm{t}\mathrm{o}\mathrm{t}\mathrm{a}\mathrm{l}}=\sum_{i=1}^{n}\sum_{t=1}^{k}{\left({X}_{it}-{\overline{X} }_{\cdot \cdot }\right)}^{2}$$

The residual error sum of squares is then

11 $${SS}_{error}=\sum_{i=1}^{n}\sum_{t=1}^{k}{({X}_{it}-{\overline{X} }_{i\cdot }-\left({\overline{X} }_{\cdot t}-{\overline{X} }_{\cdot \cdot }\right))}^{2}$$

The corresponding mean squares are

12 $$M{S}_{\mathrm{i}\mathrm{n}\mathrm{d}\mathrm{i}\mathrm{v}\mathrm{i}\mathrm{d}\mathrm{u}\mathrm{a}\mathrm{l}}=\frac{S{S}_{\mathrm{i}\mathrm{n}\mathrm{d}\mathrm{i}\mathrm{v}\mathrm{i}\mathrm{d}\mathrm{u}\mathrm{a}\mathrm{l}}}{n-1}$$

and

13 $$M{S}_{time}=\frac{S{S}_{time}}{n(k-1)}$$

14 $${MS}_{error}=\frac{1}{\left(n-1\right)*(k-1)}*S{S}_{error}$$

From these terms, the one-way ICCs are calculated as

15 $$ICC\left(\mathrm{1,1}\right)=\frac{M{S}_{individual}-M{S}_{time}}{M{S}_{individual}+\left(k-1\right)M{S}_{time}}$$

and

16 $$ICC\left(1,k\right)=\frac{M{S}_{individual}-M{S}_{time}}{M{S}_{individual}}$$

These coefficients represent the reliability of a single measurement and the average of both measurements, respectively. Note that in the original publication from Shrout & Fleiss (1979), the $S{S}_{\mathrm{i}\mathrm{n}\mathrm{d}\mathrm{i}\mathrm{v}\mathrm{i}\mathrm{d}\mathrm{u}\mathrm{a}\mathrm{l}}$ was $S{S}_{\mathrm{b}\mathrm{e}\mathrm{t}\mathrm{w}\mathrm{e}\mathrm{e}\mathrm{n}}$ and $S{S}_{time}$ was $S{S}_{\mathrm{w}\mathrm{i}\mathrm{t}\mathrm{h}\mathrm{i}\mathrm{n}}$ (the same applies to MS). To adhere to the index i (individual) and t (time) introduced above, the nomenclature was adopted for congruency.

For the two-way ICCs, the application separates participant variance, measurement variance, and residual error variance. Using these quantities, the two-way random-effects absolute-agreement ICCs are calculated as

17 $$ICC\left(2,1\right)=\frac{M{S}_{\mathrm{i}\mathrm{n}\mathrm{d}\mathrm{i}\mathrm{v}\mathrm{i}\mathrm{d}\mathrm{u}\mathrm{a}\mathrm{l}}-M{S}_{\mathrm{e}\mathrm{r}\mathrm{r}\mathrm{o}\mathrm{r}}}{M{S}_{\mathrm{i}\mathrm{n}\mathrm{d}\mathrm{i}\mathrm{v}\mathrm{i}\mathrm{d}\mathrm{u}\mathrm{a}\mathrm{l}}+\left(k-1\right)M{S}_{\mathrm{e}\mathrm{r}\mathrm{r}\mathrm{o}\mathrm{r}}+\frac{k\left(M{S}_{\mathrm{t}\mathrm{i}\mathrm{m}\mathrm{e}}-M{S}_{\mathrm{e}\mathrm{r}\mathrm{r}\mathrm{o}\mathrm{r}}\right)}{n}}$$

and

18 $$ICC\left(2,k\right)=\frac{M{S}_{\mathrm{i}\mathrm{n}\mathrm{d}\mathrm{i}\mathrm{v}\mathrm{i}\mathrm{d}\mathrm{u}\mathrm{a}\mathrm{l}}-M{S}_{\mathrm{e}\mathrm{r}\mathrm{r}\mathrm{o}\mathrm{r}}}{M{S}_{\mathrm{i}\mathrm{n}\mathrm{d}\mathrm{i}\mathrm{v}\mathrm{i}\mathrm{d}\mathrm{u}\mathrm{a}\mathrm{l}}+\frac{M{S}_{\mathrm{t}\mathrm{i}\mathrm{m}\mathrm{e}}-M{S}_{\mathrm{e}\mathrm{r}\mathrm{r}\mathrm{o}\mathrm{r}}}{n}}$$

The corresponding two-way mixed-effects consistency ICCs are

19 $$ICC\left(3,1\right)=\frac{M{S}_{\mathrm{i}\mathrm{n}\mathrm{d}\mathrm{i}\mathrm{v}\mathrm{i}\mathrm{d}\mathrm{u}\mathrm{a}\mathrm{l}}-M{S}_{\mathrm{e}\mathrm{r}\mathrm{r}\mathrm{o}\mathrm{r}}}{M{S}_{\mathrm{i}\mathrm{n}\mathrm{d}\mathrm{i}\mathrm{v}\mathrm{i}\mathrm{d}\mathrm{u}\mathrm{a}\mathrm{l}}+\left(k-1\right)M{S}_{\mathrm{e}\mathrm{r}\mathrm{r}\mathrm{o}\mathrm{r}}}$$

and

20 $$ICC\left(3,k\right)=\frac{M{S}_{\mathrm{i}\mathrm{n}\mathrm{d}\mathrm{i}\mathrm{v}\mathrm{i}\mathrm{d}\mathrm{u}\mathrm{a}\mathrm{l}}-M{S}_{\mathrm{e}\mathrm{r}\mathrm{r}\mathrm{o}\mathrm{r}}}{M{S}_{\mathrm{i}\mathrm{n}\mathrm{d}\mathrm{i}\mathrm{v}\mathrm{i}\mathrm{d}\mathrm{u}\mathrm{a}\mathrm{l}}}$$

These calculations correspond to the standard ANOVA-based ICC definitions and are consistent with the formulations used in established reliability literature (Koo & Li, 2016) and software implementations. To improve interpretability, the application also provides 95% confidence intervals for all ICCs. For ICC(1,1), ICC(1,k), ICC(2,1) and ICC(2,k), ICC(3,1), and ICC(3,k), the intervals are derived from the relevant F-ratio, transformed into ICC bounds, and are calculated as in the “irr” package (Gamer & Lemon, 2019).

### Cronbach’s alpha (α)

A further, related quantification oftentimes used for internal consistency across the two repeated measurements is Cronbach’s α (Cronbach, 1951). For two repeated measurements, α was computed from the item-variance formulation

21 $$\alpha =\frac{k}{k-1}\left(1-\frac{\sum_{j=1}^{k}{\sigma}_{t}^{2}}{{\sigma}_{total}^{2}}\right)$$

where k=2, ${\sigma}_{j}^{2}$ denotes the variance of measurement j, and ${\sigma}_{total}^{2}$ denotes the variance of the summed score across both measurements (Bland & Altman, 1997).

### Calculation of the standard error of measurement (SEoM) and minimal detectable change (MDC)

The SEoM (also oftentimes referred to as SEM (Harvill, 1991; Weir, 2005)) quantifies the expected magnitude of measurement error in the original unit of measurement, including the ICC in its calculation. In the presented work, the nomenclature is slightly adjusted to SEoM, as the abbreviation of SEM is also used for structural equation modeling). In the present application, the SEoM (Harvill, 1991; Warneke et al., 2025a) is calculated as

22 $$SEoM=S{D}_{pooled}\cdot \sqrt{1-ICC}$$

Based on the SEoM, the MDC (Seamon et al., 2022) at the 95% confidence level is then calculated as

23 $$MD{C}_{95}=1.96\cdot \sqrt{2}\cdot SEoM$$

The SEoM reflects the typical random measurement error, whereas the MDC indicates the minimum change required to exceed measurement error with 95% confidence. This logic is closely linked to the interpretation of real change in repeated-measures designs.

As outlined above, excellent reliability (e.g., ICC ≥ 0.9) (Koo & Li, 2016) can be accompanied by meaningful measurement errors with a test–retest mean and maximum absolute test–retest error of 20% and 90%, respectively (Atkinson & Nevill, 1998; Lamb, 1998; Warneke et al., 2025a). Accordingly, several authors requested the supplementation of common reliability coefficients with an agreement analysis (i.e., how close is the agreement between two measurements within a subject on the same scale) to improve interpretability and to better account for the different sources of measurement errors (Atkinson & Nevill, 1998; Barnhart et al., 2007; Warneke et al., 2025a).

## Agreement analysis in reliability research

The BA analysis evaluates the closeness of agreement between two measurements under identical conditions within a subject (Doğan, 2018; Giavarina, 2015; Haghayegh et al., 2020) rather than providing a correlation-based analysis as a stand-alone quantification (Bland & Altman, 1995, 1999; Bland & Altman, 1986; Doğan, 2018; Lin, 1989; Vetter & Schober, 2018). Therefore, BA analyses are an important complement to typical reliability analyses. While initially presented to compare a gold standard method against a novel measurement device (validity studies), it can also be used in a comparative way without having a gold standard to explore the test–retest error distribution (Bland & Altman, 1986). Agreement is evaluated by plotting the test–retest difference (*y*-axis) against the respective mean of the two measurements (*x*-axis). It graphically illustrates random scattering (random error) around the test–retest mean bias (systematic error) (Bland & Altman, 1986; Bogdanov et al., 2025; Warneke et al., 2025a). If the mean of all test trials is larger than the mean of the retest trials, the mean bias line diverges from the zero-line and indicates a systematic error. The significance of this deviation can be tested via a paired-samples *t* test (see systematic error). If the summed random scattering approaches 0, no systematic error occurred. Note, however, that the result of this test – as with other assumption tests – is sample-size dependent. Hence, the significance of this test should not be blindly used as a sole criterion to evaluate bias without visual inspection.

**Fig. 2:**
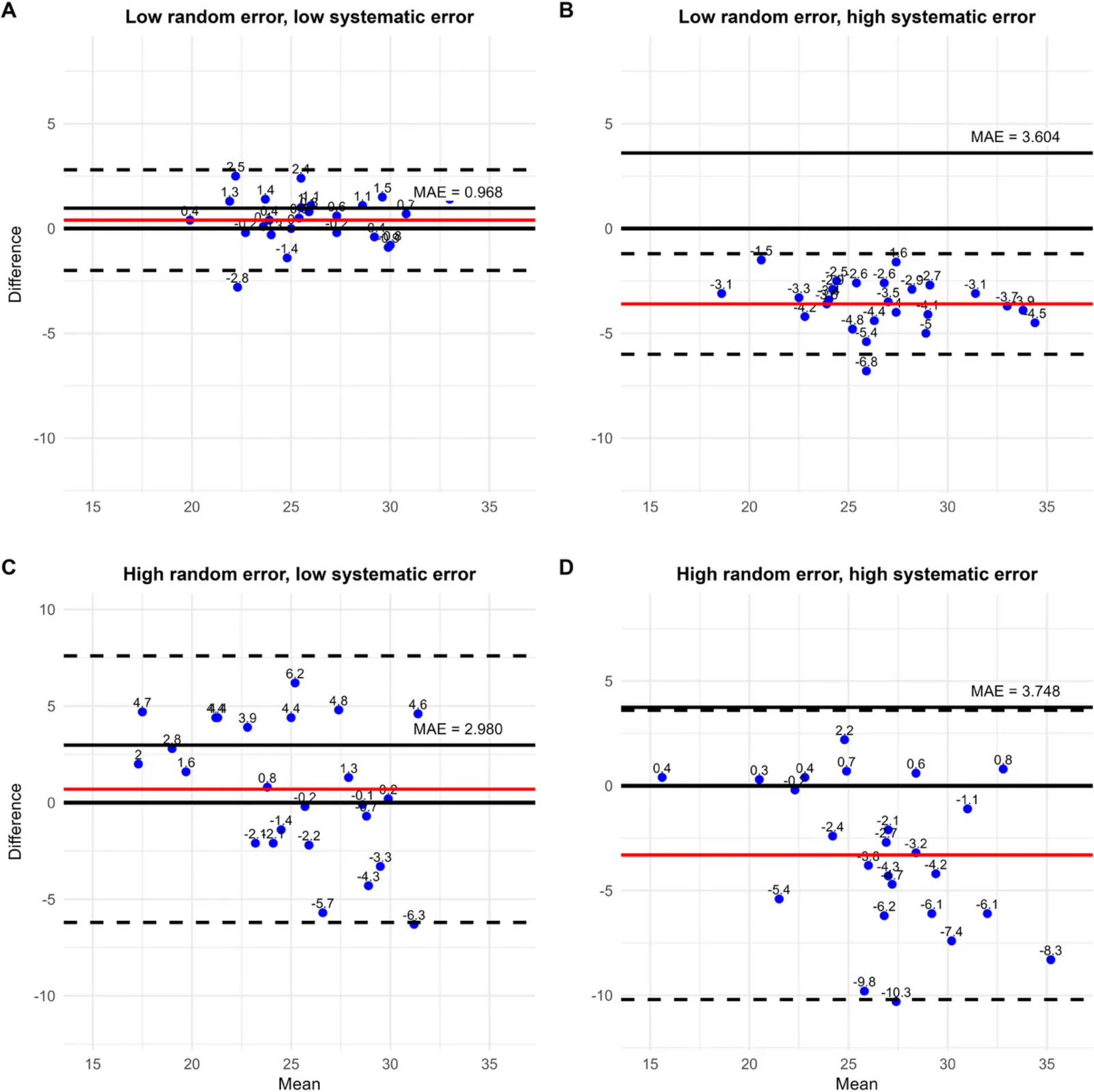
BA plots for different conditions. The *red line* illustrates the systematic bias (i.e., the average difference in the difference between the first and second test scores). The unlabeled *continuous black line* illustrates the 0 line (i.e., no difference between the two test scores), the continuous black line with the label "MAE" illustrates the Mean Absolute Error. The *dotted lines* represent the limits of agreement (LoA). The wider the LoAs, the greater the magnitude of measurement error. **A** Accurate measurement with small random error and no systematic error. **B** How missing accuracy is due to systematic error, while the random error remained small. **C** Large random error, but negligible systematic error. **D** Large random and large systematic error; thus, the worst-case scenario

The random error is commonly quantified by plotting the 95% limits of agreement (LoA) reflecting the $mean differences \pm 1.96\times Standard deviation$, which provides a reference interval for test–retest differences expected for 95% of individuals of the analyzed population (Wright & Royston, 1999). According to Barnhart et al. (2007), the agreement analysis is therefore able to account for precision and relative accuracy. If individual data points are widely scattered without a systematic mean bias, test–retest reliability is hampered by imprecision. If the systematic error diverges significantly from the zero-line, an additional systematic error suggests an accuracy problem. An accuracy problem is indicated if the retest systematically lies above or below the test, which could be attributed to habituation effects, even if precision was sufficient. In that case, the retest precisely missed hitting the correct spot. Bland–Altman plots corresponding to target shooting (see Fig. 1) are provided in Fig. 2. Underlying simulated data are provided in the Supplemental Material (Tables S1 and S2).

Before calculating the ICC, Bartlett & Frost (2008) suggested performing a BA analysis as a precondition for the validity of reliability coefficients that assume parallel readings. Here, it is noteworthy that large ICCs do not automatically refer to high accuracy (Fig. 2A) or precision (Fig. 2B), underscoring the call for supplementing ICCs via these analyses (Atkinson & Nevill, 1998).

### Calculation of Bland–Altman metrics (systematic bias, limits of agreement)

As defined above, ${d}_{i}$ (4) reflects the individual test–retest deviation, whereas the value ${m}_{i}$ (5) serves as the horizontal coordinate in the BA plot. The systematic bias is assessed by the mean of the paired differences,

24 $$\overline{d}=\frac{1}{n}\sum_{i=1}^{n}{d}_{i}$$

where a paired-samples *t* test can examine if the mean difference differs significantly from zero. The standard deviation of the paired differences is

25 $$S{D}_{d}=\sqrt{\frac{\sum_{i=1}^{n}({d}_{i}-\overline{d }{)}^{2}}{n-1}}$$

The corresponding *t*-statistic is

26 $$t=\frac{\overline{d}}{S{D }_{d}/\sqrt{n}}$$

A significant result indicates that one measurement tends to be systematically higher or lower than the other. This distinction is important because large relative reliability can still coexist with meaningful systematic bias (Warneke et al., 2025a). The LoAs are calculated as

27 $$Lo{A}_{lower}=\overline{d }-1.96\cdot S{D}_{d}$$

and

28 $$Lo{A}_{upper}=\overline{d }+1.96\cdot S{D}_{d}$$

## How to appropriately interpret Bland–Altman plots

Given the nature of BA analyses, LoAs should be assessed relative to the respective mean value. The larger the *y*-axis range in a BA plot, the lower the precision of the measurement. Reporting LoAs without referring to the mean of the measurement values can lead to misleading conclusions (Grgic et al., 2020). For instance, visual vertical perception is clinically relevant in subacute stroke patients. In one study (Piscicelli et al., 2015), BA analyses were performed between the test–retest values. The authors reported LoAs were between – 1.03 and 1.33°. Without providing a reference mean for the random error, the result is difficult to interpret. For instance, if the mean was 0.5°, this would refer to a large measurement error surpassing 100%. However, if the mean was 180°, the measurement error would be less than 1%. Although lower and upper LoAs allow for quantification, several articles miscommunicate BA analyses and LoAs and state that sufficient reliability was reached as most data points were within the LoAs (for instance: (Brandl et al., 2024b; Palsma et al., 2025), or provide LoA quantification without a mean reference, which does not allow for adequate interpretability.

Since BA analysis plots the mean differences between two values, LoAs must be interpreted in relation to the *reference mean* of the test–retest. A more appropriate reporting practice is expressing the upper and lower LoAs in relation to the respective mean (Cataldi et al., 2022). Another possibility to receive information about the measurement error is the reporting of the mean absolute error (MAE) (Willmott & Matsuura, 2005), the mean absolute percentage error or the adjusted MAPE (MAPE_adj_) (Warneke et al., 2025e). An alternative is to report the adjusted mean absolute percentage error (MAPE) (Warneke et al., 2025e), which is calculated as follows:

The MAE reflects the average absolute test–retest deviation in the original unit of measurement and is a straightforward and interpretable summary of average error magnitude. It is defined as

29 $$MAE=\frac{1}{n}\sum_{i=1}^{n}\mid {d}_{i}\mid$$

Since the MAE must be reported in combination with the respective measurement mean, the mean absolute percentage error (MAPE) can be calculated as a dimensionless quantification

30 $$MAPE=\frac{100}{n}\sum_{i=1}^{n}\left|\frac{{d}_{i}}{{X}_{i1}}\right|.$$

Since the MAPE in its original form depends on the reference mean (here ${X}_{i1}$) (as above: i stands for individual and t stands for time, and in this formula, t=1), the adjusted MAPE can be used to provide a potential solution, which is calculated based on the measurement error calculations in BA analyses. Warneke et al. (2025e) proposed:

31 $${MAPE}_{adj}=\frac{100}{n}\sum_{i=1}^{n}\left|\frac{{d}_{i}}{{m}_{i}}\right|.$$

Accordingly, MAPE expresses the average absolute test–retest deviation relative to the subject-wise mean and reports it as a percentage. This can facilitate comparisons across variables with different units or scales.

## Practice effects, reliability, and agreement analyses

In the previous sections, we outlined that practice/habituation effects result in a significant test–retest improvement in numerous cognitive tasks, such as the Eriksen flanker test, the Stroop test, Digit Span Test, or the Trail Making Test, without performing an intervention between trials. Donath et al. (2017) showed that habituation improves reliability coefficients in executive function tests, and our group demonstrated that test practicing reduced the variability of cognitive performance over time, and systematic task habituation effects can be accompanied by a trial-to-trial reduction in the random error (reference included after acceptance to keep blinding). Therefore, measurement errors are not automatically independent as assumed in the CTT second assumption, and the random error is influenced by biological variability as well as practice effects. Warneke et al. (2025c, d) repeated commonly performed neurocognitive tests twice a day for five consecutive days. Beyond the systematic bias, testing neurocognitive function (Trail Making Test, Stroop test, Choice reaction task) over five consecutive days twice per session started with trial to trial random errors of up to 6–43%, but stabilized below 5% in reaction tasks, 5–9% in interference inhibition and about 10% in visual scanning (Warneke et al., 2025c, d).

Standard habituation procedures with only one practicing appointment before data collection started (if any exist) or performing some test trials before the main testing session started are often insufficient to produce reliable and valid testing results (White et al., 2019). Accordingly, more rigorous testing protocols in neurocognitive tests that take sex- (Reimers & Maylor, 2006; Warneke et al., 2025c), age (Ramos-Loyo et al., 2022; Warneke et al., 2025d) and task specific habituation courses with a critical dependence on previous task exposure (Collie et al., 2003; Estevis et al., 2012) into account are needed. Although there are several articles that assumed their tests were reliable, per se, the question of how many habituation sessions are sufficient to reach satisfying reliability is likely context- and population-specific (Warneke et al., 2025b). As early as 1992, Thompson (1992) criticized the common practice of assuming that a test is reliable per se as "sloppy speaking". The previously outlined results underscore that sufficient task habituation to improve test–retest reliability, repeatability (closeness of agreement), and accuracy of the test critically depends on the evaluated population and should specifically be determined for a given person or sample. As part of this paper, we developed an R-based software tool that will help with the evaluation of habituation effects and whether enough practice trials were implemented before the actual testing started.

## Advanced statistical approaches to improve transparency and interpretability of reliability metrics

Atkinson & Nevill (1998) noted more than 25 years ago: *“While statistics will never be more important than a well-designed reliability study itself, it is sensible that there should be a standardized statistical analysis for any reliability study involving ratio of interval measurements”* (p. 235). For reliability studies, but also for cross-sectional and longitudinal studies, reporting repeatability and measurement errors within the construct of test–retest reliability is a cornerstone in high-quality results reporting, as pointed out in the APA reporting standards for quantitative research (Appelbaum et al., 2018). As a viable supplement, and to evaluate whether the parallel readings assumption used to justify ICC calculations is met (Bartlett & Frost, 2008), cognitive and psychological research should add agreement analyses to ensure the precision and accuracy of data collection and transparent results reporting to the reader, in addition to common reliability coefficient-based reliability metrics.

However, in its current form, BA analyses are descriptive, without content-related classification (Bland & Altmann, 1986; Giavarina, 2015) and do not allow for statistical inference statements about whether sufficient repeatability was achieved and if the measurement errors are within an acceptable range. BA analyses simply provide a post hoc description of measurement errors. To classify reliability as satisfying, acceptable limits must be defined a priori and consider clinical and practical requirements on accuracy (Giavarina, 2015; Warneke et al., 2025d). In this work, we provide a tool to pre-set acceptable limits of acceptance (LoAcc) that differ from limits of agreement, implemented in a freely available R-based tool. This tool may help researchers to facilitate a comprehensive and standardized measurement error reporting across various research disciplines, including medicine, social sciences, and empirical natural sciences.

## Establishing limits of acceptance (LoAcc) to improve interpretation

**Fig. 3:**
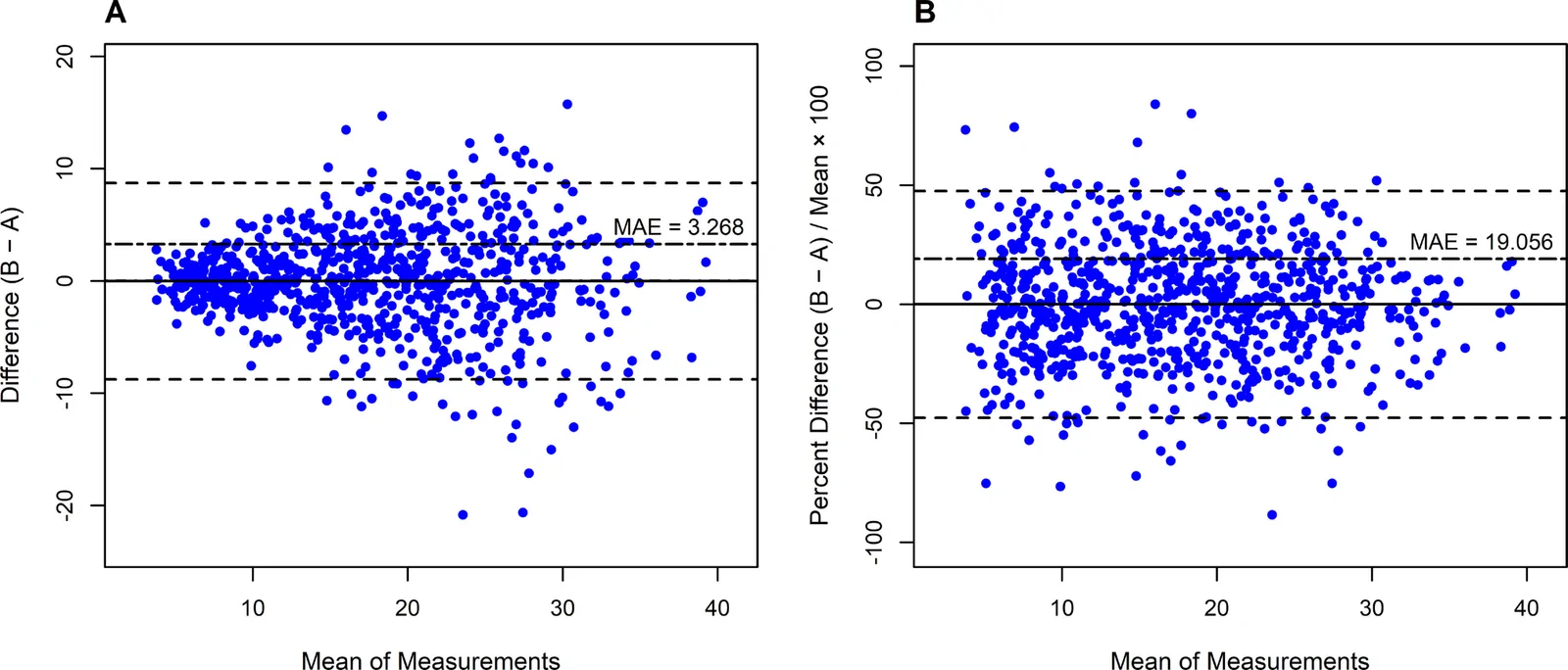
**A** Increasing test–retest difference with increasing test–retest mean while showing a constant percentage error. **B** Constant test–retest difference in dependence on the test–retest mean, which refers to an increased percentage error for smaller test values

Bland and Altman (1986) stated that *“Ideally, it should be defined in advance to help in the interpretation of the method comparison and to choose the sample size.”* (p.308). Accordingly, a content-based a priori limit determination is needed to evaluate whether sufficient reliability was reached in the data collection (Giavarina, 2015; Warneke et al., 2025a). The limitation of using parallel LoAs for small and large mean values within one analysis is explored in Fig. 3. In many empirical research areas, the measurement scattering around the mean bias gets larger when the value of the measure increases (Hopkins, 2000; Nevill & Atkinson, 1997). However, LoAs are parallel. This misleadingly suggests a constant measurement error across the sample. In fact, in their current form, parallel lines in BA analyses for test–retest differences in dependency on their respective means tolerate higher percentage measurement errors for smaller means (left side of the BA plot) compared to larger means (left side of the BA plot). In other words, the parallel LoAs suggest accepting a larger percentage error for small test–retest mean values. This complicates the interpretation and ad hoc definition of content-based acceptable measurement ranges, as LoAs being parallel to the mean bias neglects percentage errors and assumes homoscedasticity (Bartlett & Frost, 2008). Both scenarios are illustrated in Fig. 3 (A and B).

A sole post hoc description of measurement errors with parallel LoAs stands in conflict with the idea of a priori and content-based precision and accuracy classifications to define which measurement errors (systematic and random errors) can be considered acceptable. Although individual studies have predefined acceptable LoAs (Taule et al., 2023), these were 1) only set to 2±SD of the mean difference without a content-related justification (automatically, 1.96×SD are satisfying) and 2) did not account for heteroscedasticity, a phenomenon in which most data points were within these predefined limits.

In contrast, we suggest that expected percentage intervention-induced changes can serve as an orientation for which random measurement error can be considered appropriate to achieve sufficient precision. A systematic error is never acceptable for accurate measurements (and hampers the validity of the ICC (Bartlett & Frost, 2008)). This may vary depending on the goals and scientific field, with some areas probably requiring narrower acceptable measurement error than others. This approach can substitute abstract reliability coefficients such as ICC or Cronbach’s α (Koo & Li, 2016) by providing content-based agreement analyses (Bland & Altman, 1986; Giavarina, 2015).

### Calculation of limits of acceptance (LoAcc)

In contrast to the LoAs (see formulas 31–32), the acceptable threshold is defined proportionally to the subject-wise mean:

32 $$LoAc{c}_{i}=\pm (p\cdot \mid {m}_{i}\mid ),$$

where p denotes the user-defined acceptable proportional error threshold and ${m}_{i}$ is the subject-wise mean of the two repeated measurements. Accordingly, a paired difference is classified as acceptable if

33 $$-p\cdot \mid {m}_{i}\mid \le {d}_{i}\le p\cdot \mid {m}_{i}\mid$$

The acceptable (absolute test–retest differences, see *y*-axis in the BA analysis) range increases with increasing measurement means, allowing the visualization and evaluation of proportional agreement limits in the presence of heteroscedasticity.

## Advancing reliability reporting

To help with practical investigation of reliability, a Shiny app will be presented to automate the calculation of key reliability metrics in the following (Access: https://cloud.uni-jena.de/s/Zf8XW62zQeyssEy or via the Open Science Framework profile (osf.io/gbcea). These include Cronbach’s α (Bland & Altman, 1997), intra-class correlation (ICC) (Koo & Li, 2016), standard error of measurement (SEM) (Palta et al., 2011; Weir, 2005), and minimal detectable change (MDC) (Cesar et al., 2021; Seamon et al., 2022) for relative and absolute reliability. A descriptive way to quantify the actual random error is through the mean absolute error (MAE) and mean absolute percentage error (MAPE) (Warneke et al., 2025a; Willmott & Matsuura, 2005), summing up the absolute test–retest differences by dividing them by the participant count in absolute terms and expressed as a percentage, respectively. Additionally, the script accounts for systematic bias by performing a paired-samples *t* test (Atkinson & Nevill, 1998).

The Shiny app enhances the interpretability of reliability metrics, so that practitioners and clinicians can easily review and interpret study results in light of the measurement error. Therefore, the script allows users to define pre-set LoAcc, providing a structured approach for evaluating acceptable measurement errors in their dataset. Both features improve the clarity of measurement error assessment and facilitate more standardized reporting practices. In the following section, we will describe in detail an example of how the script works and what formulas are used.

## LoA versus LoAcc in neurocognitive tests: An example of reliability evaluation, including reliability coefficients as well as agreement analysis

Assuming that practice or task habituation compromises the internal validity of a data set via meaningful systematic and random errors, the quantification of both error sources can be performed via BA analyses. Therefore, in the first step, an acceptable, content-based measurement error range must be defined (Bland & Altman, 1986; Giavarina, 2015; Warneke et al., 2025a). The classification of measurement errors as being clinically or practically relevant depends on a variety of factors, but pre-post effects extracted from previous studies (with comparable samples and experimental settings) could be used to define an acceptable range.

## The appropriate pre-set measurement error

A measurement error such as a coefficient of variation ≤10% (Cabarkapa et al., 2025; Luque Laguna et al., 2020) is often described as acceptable, but we propose further contextualization. For instance, if an intervention results in a pre-post change of 100%, a (random) measurement error of 10% might be acceptable. In contrast, if an intervention effect of 10% is assumed, a measurement error in the pre- and post-test of 10% is unacceptable. Therefore, authors should make this decision transparently in studies to communicate to the reader which measurement error was tolerated.

After defining a tolerable measurement error for test–retest deviations, data can be promptly analyzed. The choice of the acceptable measurement error margin strongly depends on the study, research area and goal. For instance, Jogie et al. (2025) allowed a maximum difference of ±10 in the Goal Attainment Scale and the Functional Independence Measure in the agreement analysis between different observers. In blood pressure measurements, Gibbon et al. (2022) pre-set a limit of ±5 mmHg, and Hwang et al. (2022) predefined ±2 units in the Rowland Universal Dementia Assessment Scale and the Geriatric Depression Scale.

In a previous study, Warneke et al. (2025c) showed that task-to-task habituation diminished the systematic and random error until both stabilized at a significantly reduced level. Since no generally implemented guidelines on which level of measurement error is acceptable exist, this example relies on the magnitude of MAPE observed after four days of habituation as outlined in Warneke et al. (2025c), with 4.66% MAPE. Therefore, the acceptable measurement error was set to 5% to illustrate how the Shiny app works. To illustrate, imagine a Stroop test was performed on four consecutive days as described in the latter study. Note that the user of the Shiny app can pre-set the acceptable measurement error margin to any percentage value that makes sense from a content-related perspective.

## Data structure

**Fig. 4:**
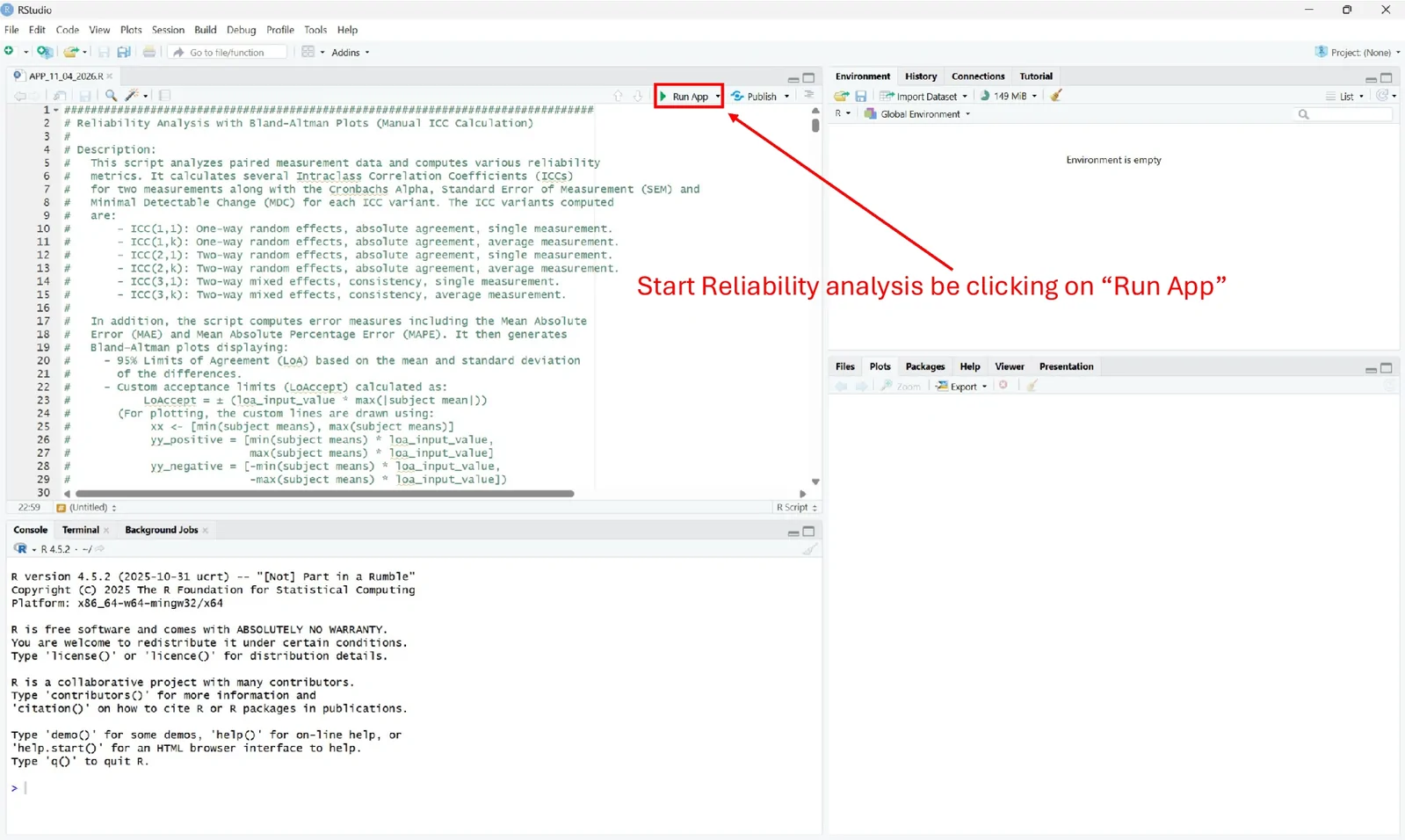
Where the user can start the app

Before running the application, the dataset must be arranged in a standardized wide format. Because the present workflow is restricted to pairwise reliability analysis, all implemented reliability indices are calculated from exactly two repeated measurements, for example, test versus retest (test–retest reliability). It can also be applied for objectivity (if one column contains values from rater 1 and the other rater 2) or validity (column 1 is the gold standard, column 2 the device that should be evaluated). To ensure error-free import and processing of the data, the first column must contain the participant identifier, whereas all subsequent columns must contain measured variables. These variables can be arranged pairwise in advance, because the script automatically treats two consecutive columns as one measurement pair. Thus, columns 2 and 3 form the first pair, columns 4 and 5 form the second pair, etc. Alternatively, measurement pairs can also be selected manually within the application (see Fig. 4). In the present example, we simulated Stroop test data that can be accessed for transparency in the Supplementary Material Excel file Data_plot2). The first column contains the participant identifier (ID), whereas each test day is represented by two separate columns containing the repeated measurements (e.g., day 1 and day 2).

## Steps through the Shiny app

The Shiny app can be started in RStudio by running the script. The script first installs and loads all required packages. Once the application window opens, the user can review the complete code used for maximal transparency and, once the data set was uploaded (see first step in the description), the user is guided through the analysis procedure step by step. Therefore, for inline code, we refer to the uploaded file, usable via the following link: https://cloud.uni-jena.de/s/Zf8XW62zQeyssEy or made accessible via our Open Science Framework profile (osf.io/gbcea). The following tutorial will guide the potential user through the application process:

*Run the application*: After copying the Code into RStudio, the user can review the individual steps of the application with some commentary and instructions on the RStudio surface. By clicking on “Run App” the analysis is initiated (see Fig. 4)

**Fig. 5:**
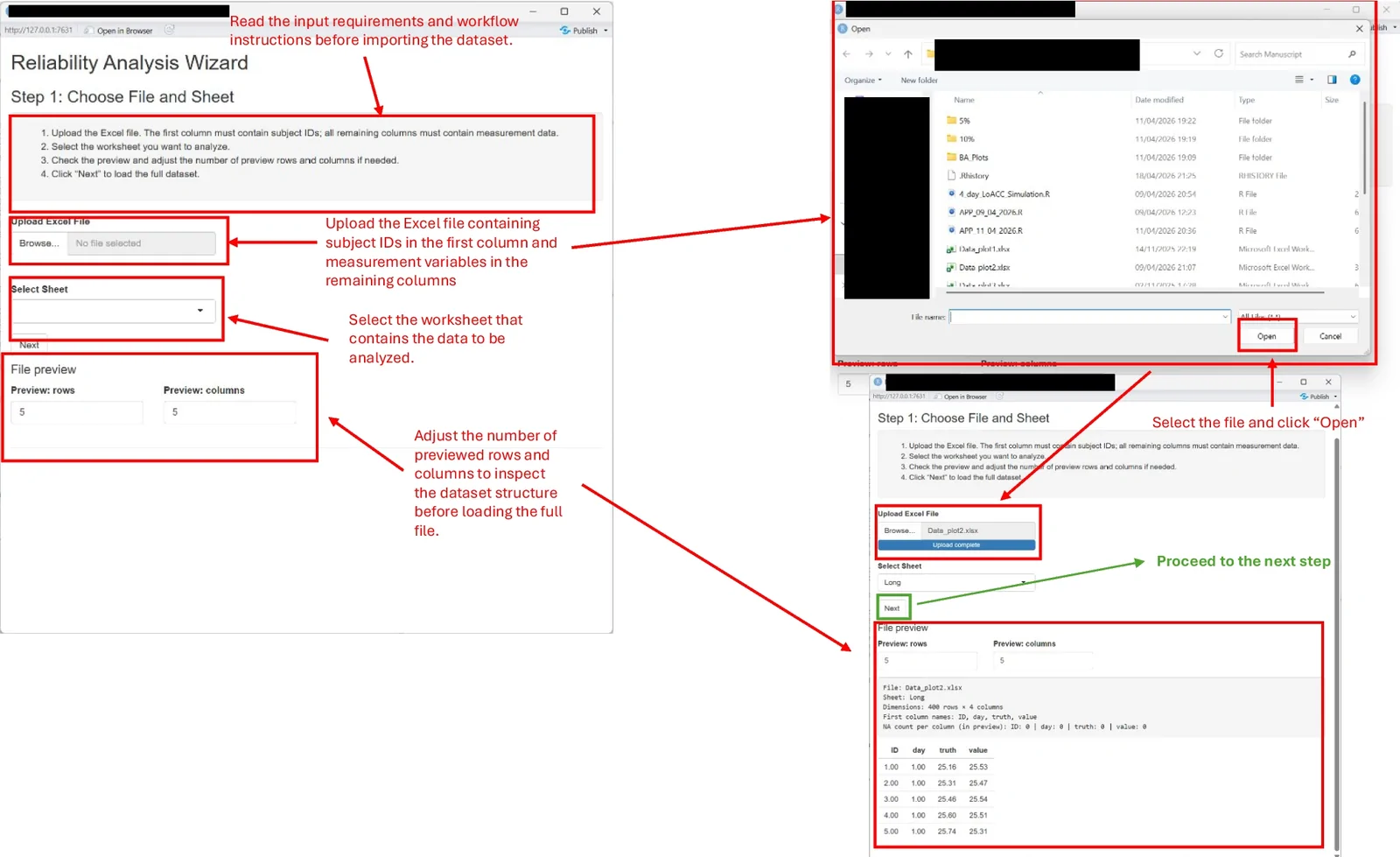
The first user surface appearing after the app was started, where an Excel file can be uploaded and where the described preview will disappear

*Upload the Excel file*: containing the data and select the relevant worksheet. To facilitate verification of the data structure, the application provides a preview of the imported file (i.e., names of the first rows and columns, file name, sheet name, dataset dimensions, and the number of missing values per column) (see Fig. 5).

**Fig. 6:**
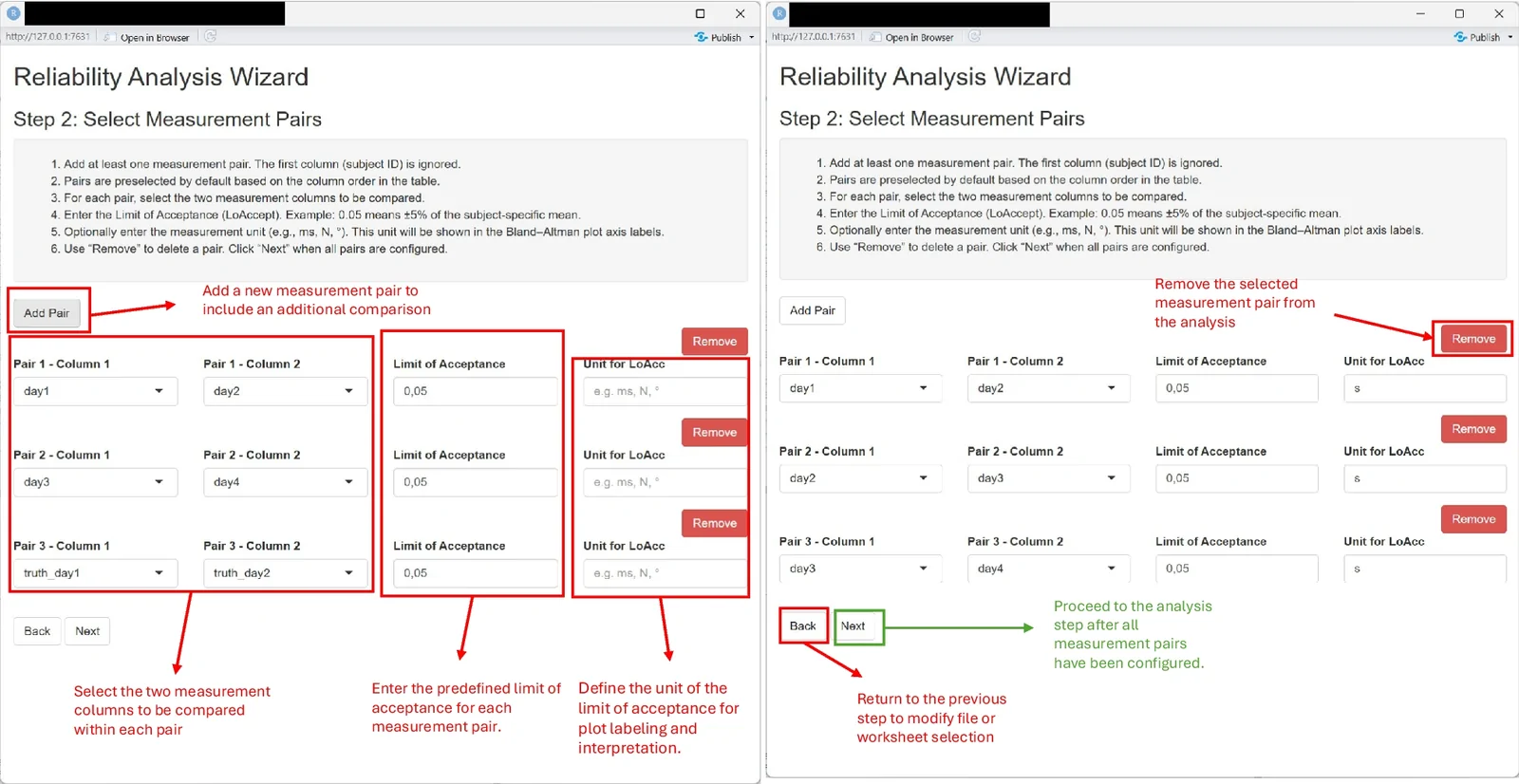
Column comparisons can be defined and added and the pre-set limits of acceptance can be defined per pair

*Define the measurement pairs*: If the variables were arranged pairwise in advance, the application can assign the corresponding column pairs automatically. The selected columns can also be modified manually. At this stage, the user additionally specifies the LoAcc for each variable pair. A default value of 0.05, corresponding to an acceptable random scattering/random error of 5% in our example. This value can be changed according to the user’s preference. The user may also optionally define the measurement unit for each variable pair (e.g., ms, *N*, °), which will then be displayed in square brackets in the *x*- and *y*-axis labels of the Bland–Altman plots (see Fig. 6).

**Fig. 7:**
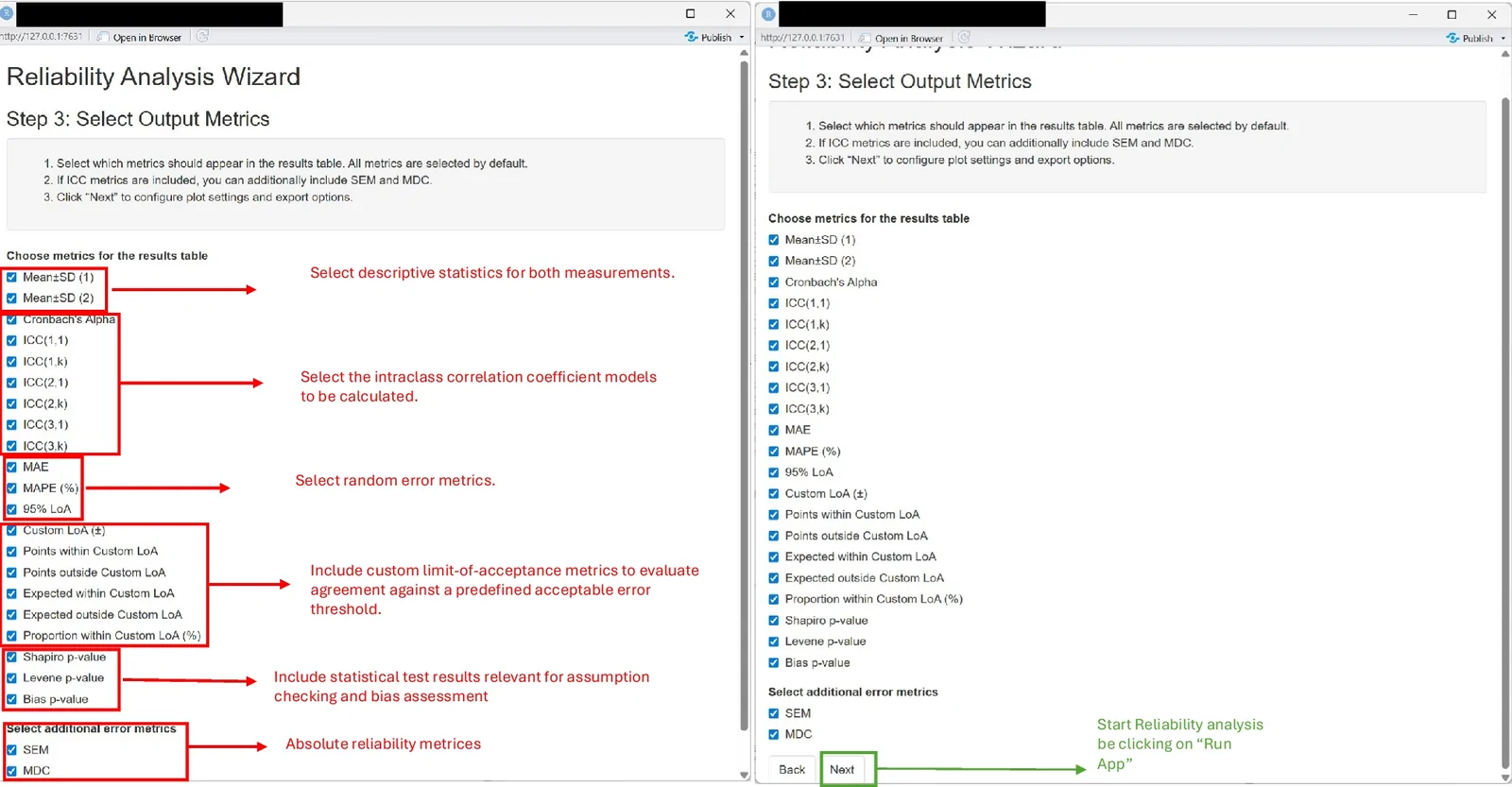
The analyses and reliability metrics can be chosen, whether all should be calculated or if the user wants to make a choice

*Select output metrics*: Based on Atkinson & Nevill (1998), our package can calculate the most common metrics used in test–retest reliability and agreement analyses such as Cronbach’s α, ICC, SEM, MDC, CV, MAE, MAPE, limits of agreement, systematic bias, among others. To account for different study and evaluation settings, all available ICCs can be evaluated with the SEM and MDC using the respective ICC version (see Fig. 7). Available quantifications and how they were calculated are described in the following section “Calculation of metrics” in detail.

**Fig. 8:**
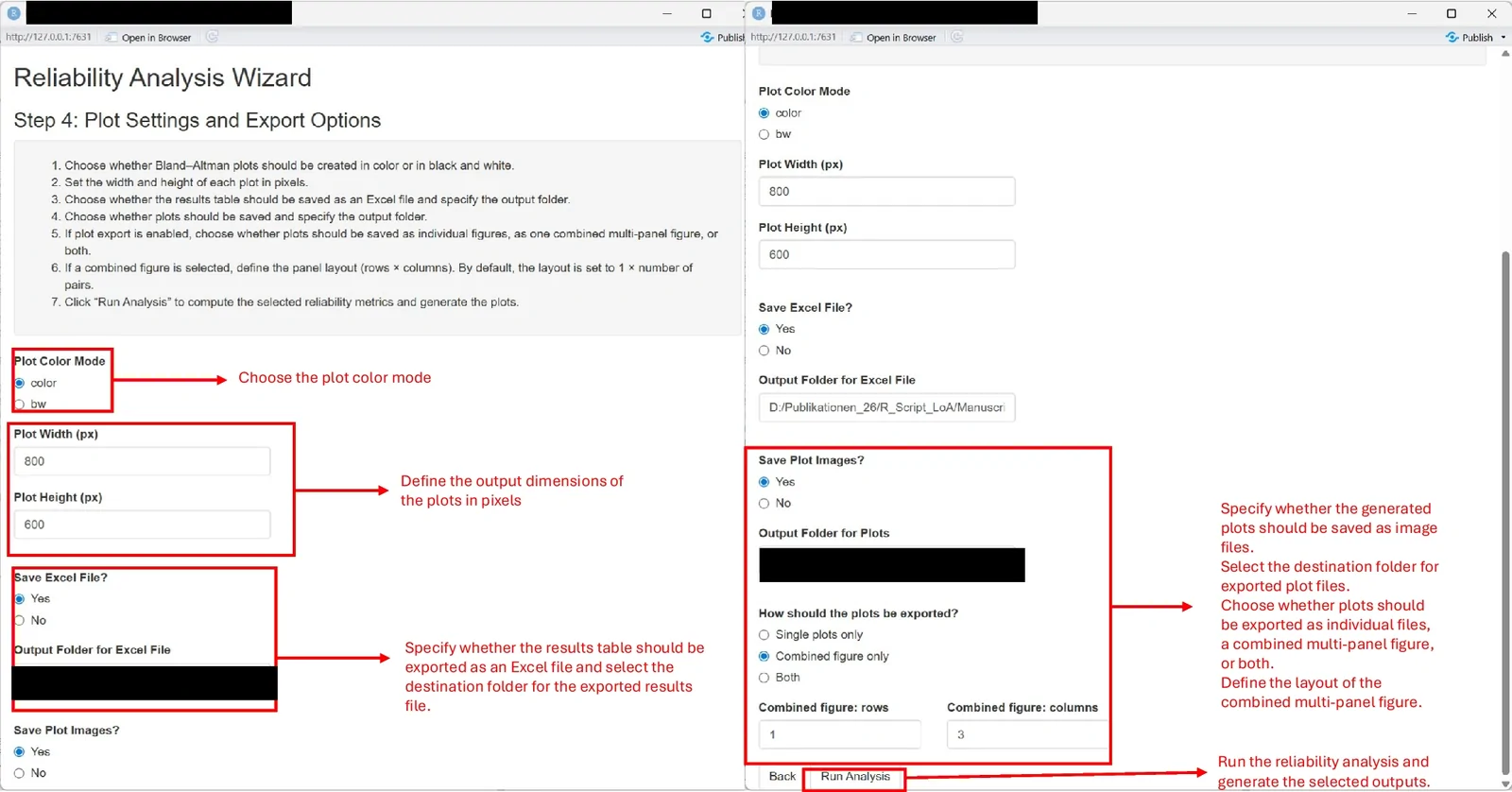
How the user will make a choice about the format of the output

*Define the export and plot settings*: These settings include the plot mode (color or black and white), the image dimensions, and the output directories for the Excel results file and the plot files. In addition, the user can define whether the Bland–Altman plots should be saved as individual figures, as one combined multi-panel figure, or in both formats. If a combined Fig. is selected, the panel layout (rows × columns) can also be specified. Default settings are pre-programmed, including a colored plot with a resolution of 800 × 600 pixels and export paths corresponding to the current working directory (see Fig. 8).

**Fig. 9:**
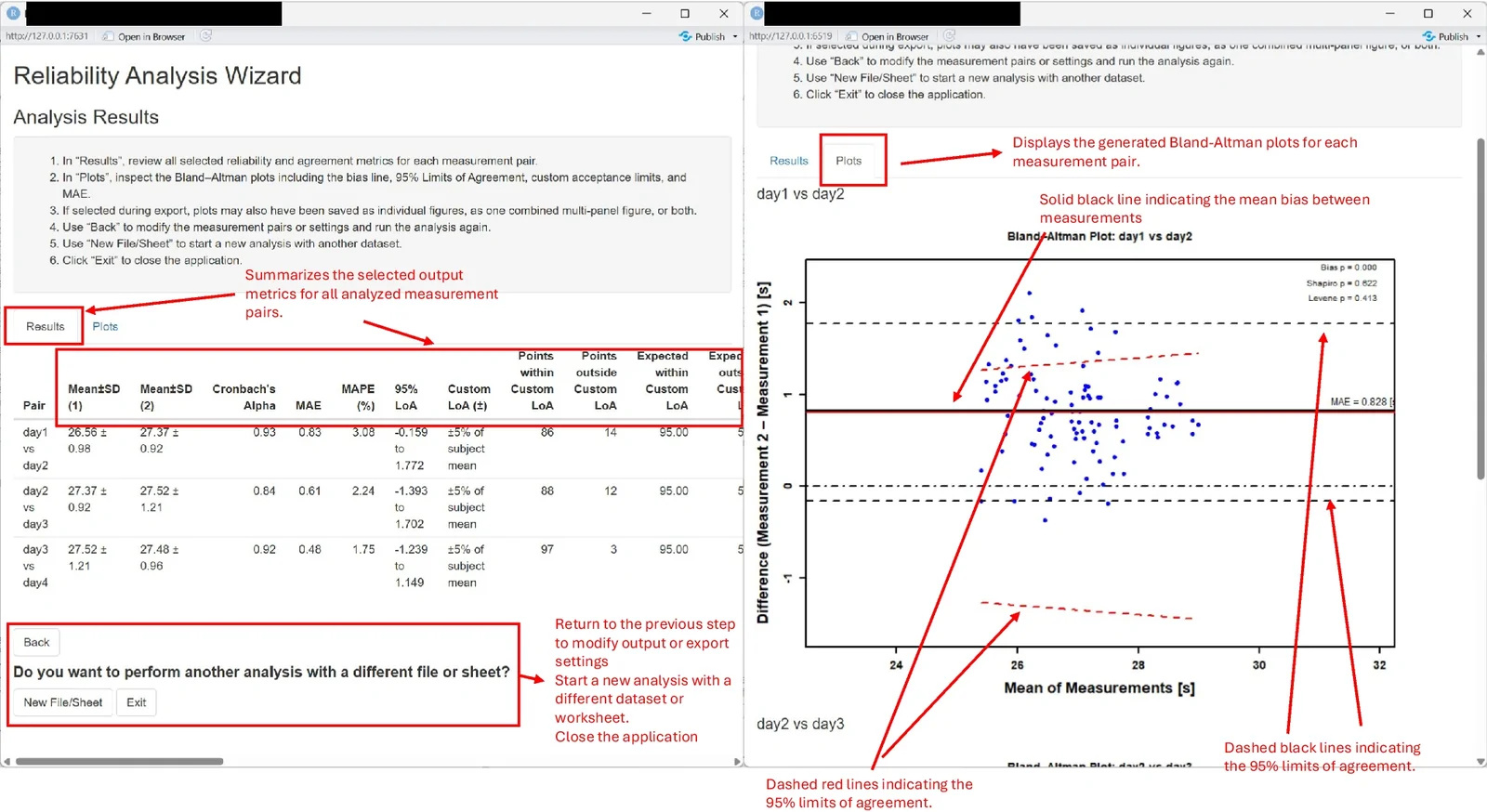
Output of the analysis, including the table as well as a Bland–Altman analysis with commonly known limits of agreement, as well as included pre-set (here 5%) limits of acceptance as well as the mean bias. Furthermore, the assumption check and paired-sampled *t* test results can be found in the *upper-right corner*, and the MAE was added in addition to the systematic bias line and quantified

*Start the analysis*: The application then displays the selected test–retest reliability metrics in tabular form and provides the corresponding BA plots within the graphical interface (see Fig. 9). In addition, the results are exported automatically. If desired, the user may subsequently return to earlier steps, modify the settings, analyze another file or worksheet, or close the application.

## Results interpretation of the data simulation outcome

To make the different scenarios visible, we simulated a data set that could illustrate Stroop color word test accomplishment times. The data were generated to illustrate an optimized task habituation quantification process to illustrate the examples. Therefore, four testing days were generated, and the test–retest analysis, including the described metrics, was performed from the initial day (day1) to the second, the second to the third, and the third to the fourth day. As previously described, the LoAcc are not displayed as parallel horizontal lines. Instead, they are adjusted to the magnitude of the respective test–retest mean, because fixed absolute limits would permit disproportionately large percentage errors at small means, which appears unreasonable. Thus, acceptable percentage test–retest differences in the BA analysis require proportional limits that increase with increasing mean values. This limit was exemplary set to 5% in this analysis.

**Fig. 10:**
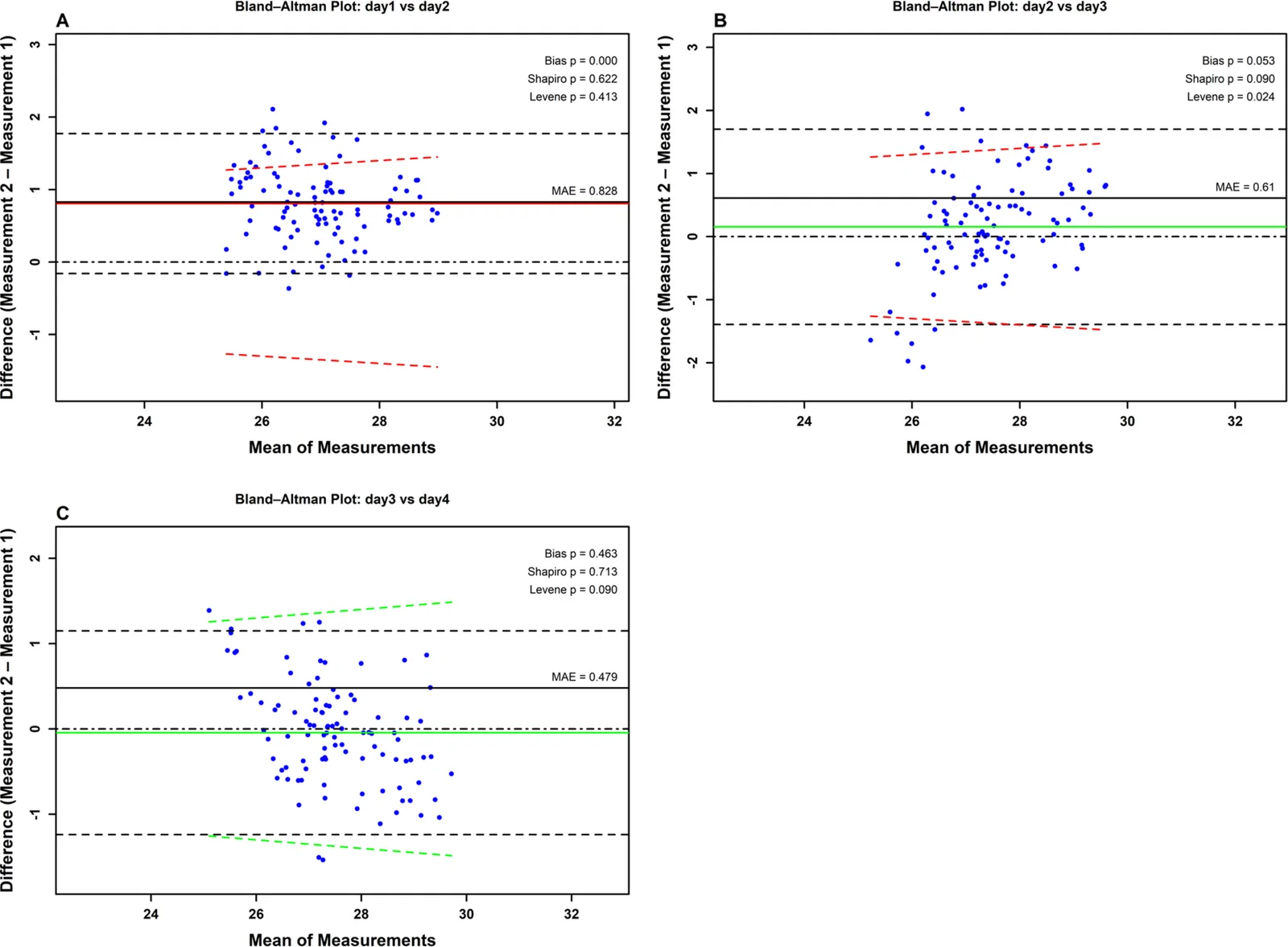
A simulated scenario of trial to trial practice/habituation effects showing (**A**) a systematic and random error (lack of accuracy) between test day 1 and 2 (mean bias line and LoAccs inked in *red*), (**B**) improved, but still insufficient random errors (red LoAccs) without a significant systematic error (green mean bias line) as an expression of task habituation between test day two and three and (**C**) sufficient accuracy, reflected in the absence of a systematic error (*green mean bias line*) and all random error test–retest differences within the LoAcc between day three and four (green LoAccs). Simulated data can be accessed via the Supplementary File “Data_plot3” via https://cloud.uni-jena.de/s/Zf8XW62zQeyssEy or our Open Science Framework profile (osf.io/gbcea).

In the comparison between day 1 and day 2 (Fig. 10A), the data indicate insufficient accuracy. Mean performance increased from 26.56 ± 0.98 to 27.37 ± 0.92, and the bias test was significant (*p* < 0.001), indicating systematic error. Consistently, the bias line is shown in red. In addition, 86 of 100 observations fell within the predefined LoAcc, although 95 of 100 would have been required to satisfy the 5% criterion. The LoAcc are therefore also displayed in red, indicating insufficient precision. The MAE was 0.828 s, and the 95% limits of agreement ranged from − 0.159 to 1.772 s. Together, these metrics indicate that both systematic error and random scatter were outside the acceptable range.

For day 2 vs. day 3 (Fig. 10B), the results suggest partial habituation. Mean performance changed only slightly from 27.37 ± 0.92 to 27.52 ± 1.21, and the bias test was no longer statistically significant (*p* = 0.053), suggesting that the systematic shift had largely disappeared; accordingly, the bias line is shown in green. However, random error remained too high, as 88 of 100 observations were within the LoAcc, so the LoAcc remained red. Although the MAE decreased to 0.610 s, the predefined precision criterion was still not met. Thus, Fig. 10B reflects improved, yet still insufficient, measurement precision. Importantly, the output provided by the R package also allows a more differentiated interpretation of such cases, because a slight deviation from the acceptance threshold may be judged differently from a substantial failure to meet the criterion.

In the comparison between day 3 and day 4 (Fig. 10C), the data fulfilled the predefined criterion for acceptable accuracy. Mean performance remained stable (27.52 ± 1.21 vs. 27.48 ± 0.96), the bias test was non-significant (*p* = 0.463), and 97 of 100 observations fell within the LoAcc. Only three observations were outside the acceptable range, which is below the tolerated number of five. The MAE decreased further to 0.479 s, and the 95% limits of agreement narrowed to − 1.239 to 1.149 s, indicating that both systematic and random error had decreased to an acceptable level. In this case, both the bias line and the LoAcc are shown in green.

The ICCs showed the same general pattern, increasing across the consecutive comparisons and thereby indicating improved stability over sessions. Similar tendencies were also observed for SEM and MDC, which were lowest in the final comparison. Overall, Fig. 10 illustrates a progression from insufficient accuracy with both systematic and random error, to partial habituation with reduced systematic error but still excessive random scatter, and finally to acceptable measurement quality once both error sources fall within the predefined limits.

It should also be noted that with progressing habituation, the conventional LoA and the LoAcc become more closely aligned, whereas in the first comparison (Fig. 10A) they differ more markedly. This can be explained by two factors. First, systematic bias shifts the data vertically, and the LoA are always distributed symmetrically around the mean bias. Second, reduced precision increases the spread of the data and thus widens the LoA. In the final comparison, the systematic error had largely dissipated, and the random scatter had decreased, resulting in a closer correspondence between LoA and LoAcc. This again illustrates that the LoA are descriptive in nature, whereas the LoAcc provide an additional content-based classification criterion defined a priori (see Fig. 10). The outcome table is provided in Table 2.

**Table 2:** Results for the automated test–retest calculation for a Stroop test, accompanying the plots in the figure. Here, four test days and 100 participants were simulated to illustrate a scenario how practice effects could be illustrated. Note that we calculated all available options of the ICC and the respective SEM and MDC here

Pair	Day 1 vs. Day 2	Day 2 vs. Day 3	Day 3 vs. Day 4
Mean ± SD (1)	26.56 ± 0.98	27.37 ± 0.92	27.52 ± 1.21
Mean ± SD (2)	27.37 ± 0.92	27.52 ± 1.21	27.48 ± 0.96
Cronbach’s alpha	0.927	0.843	0.916
MAE	0.828	0.61	0.479
MAPE (%)	3.08	2.24	1.75
95% LoA	– 0.159 to 1.772	– 1.393 to 1.702	– 1.239 to 1.149
Custom LoA (±)	±5% of subject mean	±5% of subject mean	±5% of subject mean
Points within custom LoA	86	88	97
Points outside custom LoA	14	12	3
Expected within custom LoA	95	95	95
Expected outside custom LoA	5	5	5
Proportion within custom LoA (%)	86	88	97
Shapiro *p* value	0.6221	0.09047	0.71284
Levene *p* value	0.41309	0.02406	0.08999
Bias *p* value	0	0.05343	0.46347
ICC(1.1)	0.58	0.722	0.845
ICC(1.1) 95% CI	[0.434. 0.696]	[0.614. 0.804]	[0.778. 0.893]
SEM ICC(1.1)	0.614	0.565	0.43
MDC ICC(1.1)	1.702	1.566	1.191
ICC(1.k)	0.734	0.839	0.916
ICC(1.k) 95% CI	[0.605. 0.821]	[0.761. 0.892]	[0.875. 0.943]
SEM ICC(1.k)	0.489	0.43	0.316
MDC ICC(1.k)	1.354	1.193	0.877
ICC(2.1)	0.635	0.724	0.845
ICC(2.1) 95% CI	[– 0.081. 0.872]	[0.614. 0.805]	[0.778. 0.893]
SEM ICC(2.1)	0.572	0.564	0.43
MDC ICC(2.1)	1.585	1.563	1.191
ICC(2.k)	0.777	0.84	0.916
ICC(2.k) 95% CI	[– 0.211. 0.934]	[0.761. 0.892]	[0.875. 0.943]
SEM ICC(2.k)	0.447	0.429	0.316
MDC ICC(2.k)	1.24	1.19	0.877
ICC(3.1)	0.865	0.729	0.844
ICC(3.1) 95% CI	[0.805. 0.907]	[0.622. 0.809]	[0.777. 0.893]
SEM ICC(3.1)	0.348	0.558	0.431
MDC ICC(3.1)	0.966	1.547	1.194
ICC(3.k)	0.927	0.843	0.916
ICC(3.k) 95% CI	[0.892. 0.951]	[0.767. 0.895]	[0.875. 0.943]
SEM ICC(3.k)	0.255	0.424	0.317
MDC ICC(3.k)	0.707	1.177	0.879

ICC = intraclass correlation coefficient, MDC = minimal detectable change, LoA = limits of agreement, MAE = mean absolute error, MAPE = mean absolute percentage error, SD = standard deviation, SEM = standard error of measurement

**Fig. 11.**
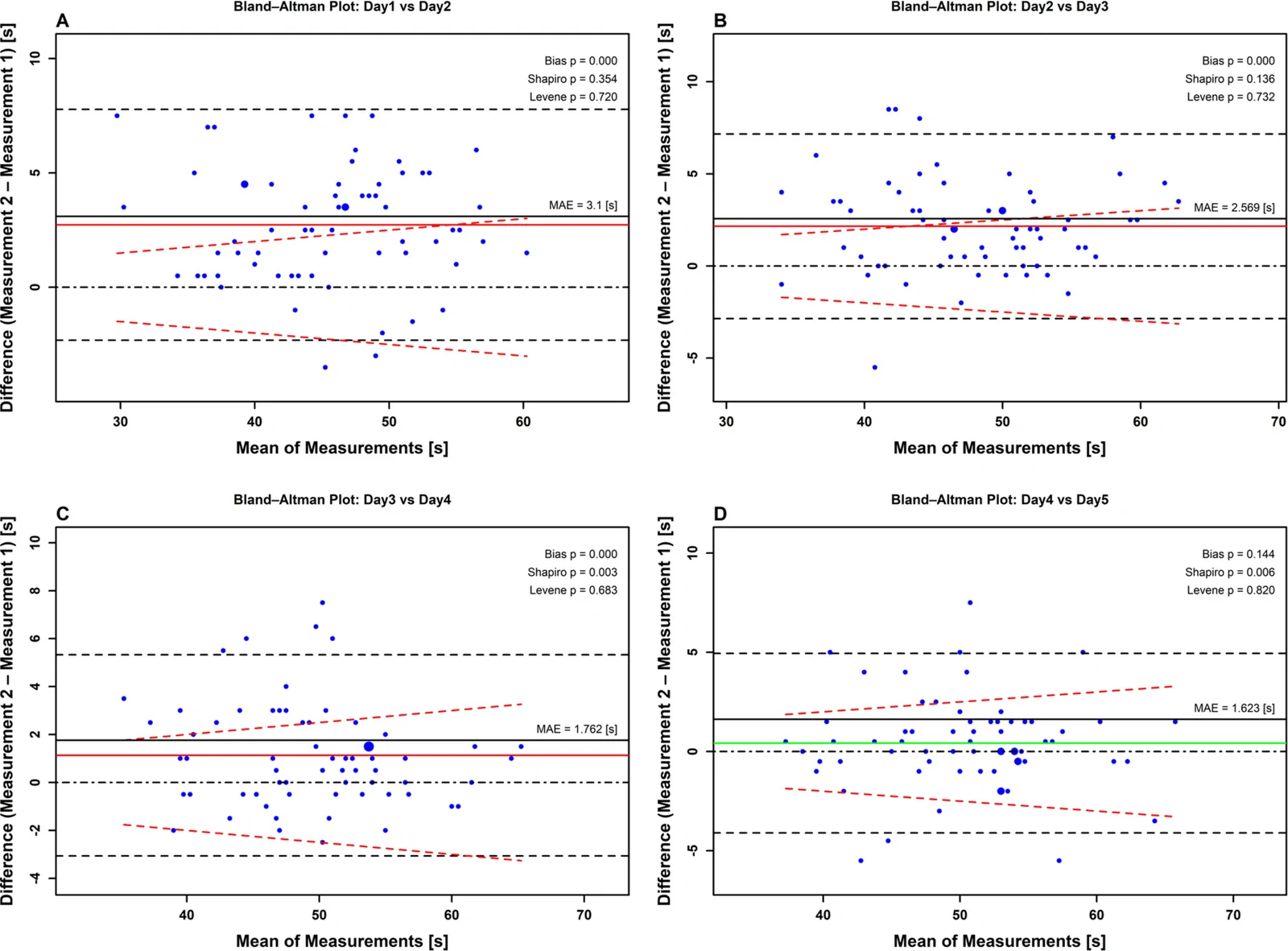
How the software application illustrates the task habituation effect applied to a data collection in the Choice reaction task exemplified for a 5% acceptable threshold. Mean bias and LoAccs are easier indicated in *red* if assumptions were unfulfilled, while in the last day 4 to day 5 comparison, the mean bias disappeared and illustrated no significant mean bias (parallel readings, see classical test theory). The original data can be accessed via the supplementary File “Data_plot4” via https://cloud.uni-jena.de/s/Zf8XW62zQeyssEy or via the OSF (osf.io/gbcea)

**Fig. 12:**
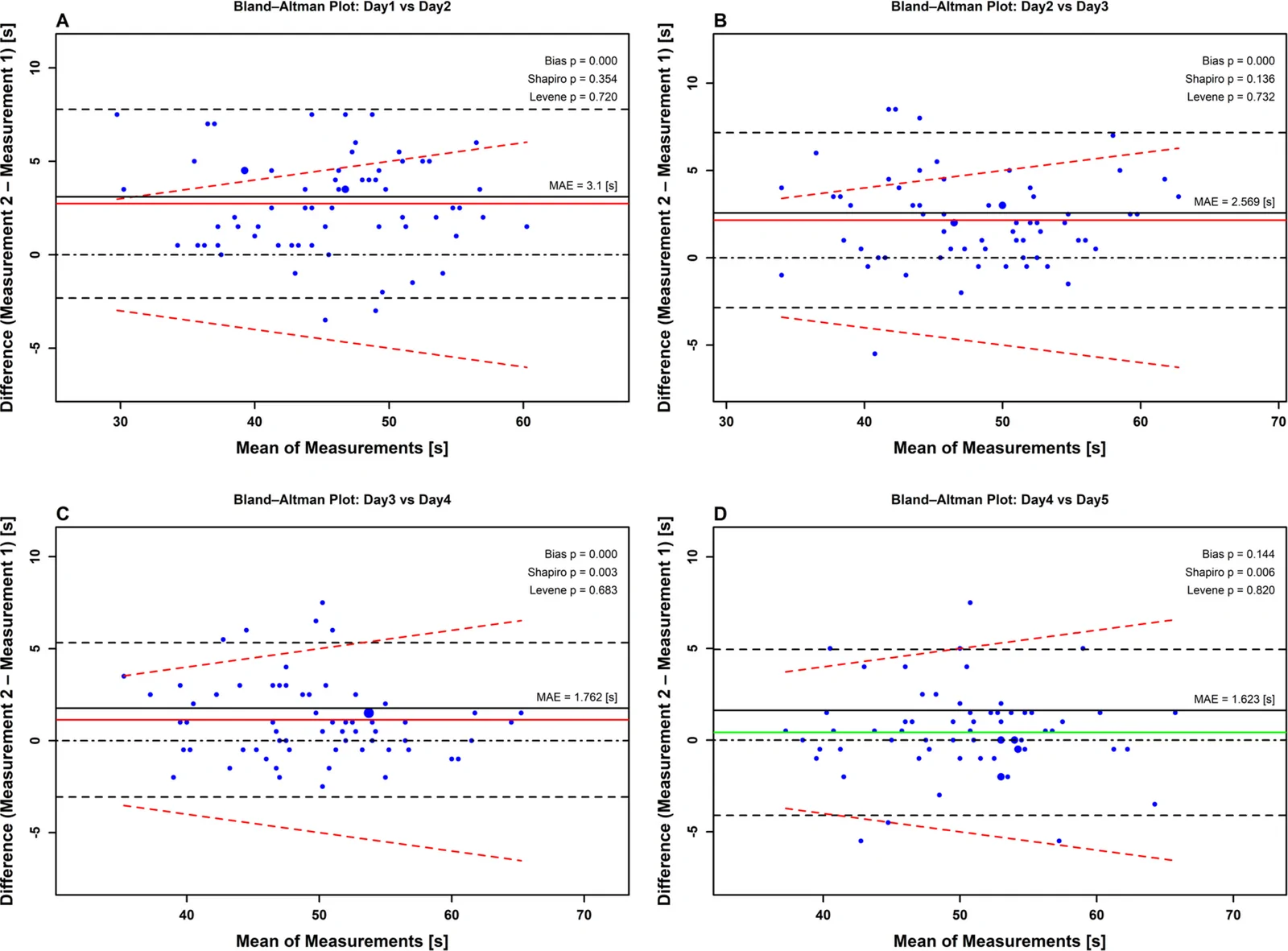
How the software application illustrates the task habituation effect applied to data collection in the Choice reaction task exemplified for a 10% acceptable threshold. Mean bias and LoAccs are easer inked in red if assumptions were unfulfilled, while in the last day 4 to day 5 comparison, the mean bias disappeared and illustrated no significant mean bias (parallel readings, see classical test theory). The original data can be accessed via the supplementary File “Data_plot4” via https://cloud.uni-jena.de/s/Zf8XW62zQeyssEy or our Open Science Framework profile (osf.io/gbcea)

To illustrate the applicability of the script for real empirical data, we applied the R script to one of our previously published datasets examining the impact of task habituation on systematic error, random error, and test–retest reliability coefficients in, among others, the choice reaction task across five testing days (Warneke et al., 2025d; Ethics No. GZ.39/163/63). To demonstrate how the predefined LoAcc influences interpretation, we analyzed the data using both a 5% and a 10% threshold (although commonly provided as the coefficient of variability), as these thresholds provide a baseline for a potential quantification, which is discussed in the literature across different disciplines (Currell & Jeukendrup, 2008; Marenco et al., 2006; Westheim et al., 2023). Whether a 5% (Fig. 11) or 10% (Fig. 12) limit is applicable might remain disputable as being arbitrary (Atkinson & Nevill, 1998), but is not the focus of the present work. The resulting plots are shown in Figs. 11 and 12, and the corresponding output for both 5% and 10% LoAccs is presented in Table 3. Notably, the 10% threshold (Fig. 12) also illustrates a near-threshold case: three data points were allowed to fall outside the LoAcc, but this criterion was exceeded by one point lying exactly on the limit. Thus, although the LoAcc are still displayed in red, the 10% threshold was almost satisfied, demonstrating that such classifications should always be interpreted in light of the magnitude and context of the deviation rather than as a purely binary decision.

**Table 3:** Results for the choice reaction task analysis, which is graphically illustrated in Figs. 5 and 6 with the 5% and 10% pre-set LoAccs scenarios

Pair	Day 1 vs. Day 2	Day 2 vs. Day 3	Day 3 vs. Day 4	Day 4 vs. Day 5
Mean ± SD (1)	44.13 ± 6.95	46.86 ± 6.86	49.02 ± 6.82	50.15 ± 6.49
Mean ± SD (2)	46.86 ± 6.86	49.02 ± 6.82	50.15 ± 6.49	50.57 ± 6.42
Cronbach’s alpha	0.964	0.964	0.974	0.967
MAE	3.1	2.569	1.762	1.623
MAPE (%)	6,97	5.56	3.72	3.28
95% LoA	– 2.317 to 7.779	– 2.855 to 7.163	– 3.064 to 5.325	– 4.102 to 4.948
5% Custom LoA (±)	±5% of subject mean	±5% of subject mean	±5% of subject mean	±5% of subject mean
5% Points within custom LoA	28	37	47	51
5% Points outside custom LoA	37	28	18	14
5% Expected within custom LoA	62	62	62	62
5% Expected outside custom LoA	3	3	3	3
5% Proportion within custom LoA (%)	43.08	56.92	72.31	78.46
10% Custom LoA (±)	±10% of subject mean	±10% of subject mean	±10% of subject mean	±10% of subject mean
10% Points within custom LoA	50	55	60	61
10% Points outside custom LoA	15	10	5	4
10% Expected within custom LoA	62	62	62	62
10% Expected outside custom LoA	3	3	3	3
10% Proportion within custom LoA (%)	76.92	84.62	92.31	93.85
Shapiro *p* value	0.35426	0.13551	0.00321	0.00645
Levene *p* value	0.71965	0.73154	0.68293	0.81955
Bias *p* value	0	0	0.00007	0.14446
ICC(1,1)	0.859	0.885	0.935	0.935
ICC(1,1) 95% CI	[0.779, 0.911]	[0.818, 0.928]	[0.896, 0.96]	[0.896, 0.96]
SEM ICC(1,1)	2.595	2.325	1.695	1.647
MDC ICC(1,1)	7.193	6.443	4.7	4.565
ICC(1,k)	0.924	0.939	0.967	0.966
ICC(1,k) 95% CI	[0.876, 0.954]	[0.9, 0.963]	[0.945, 0.98]	[0.945, 0.979]
SEM ICC(1,k)	1.903	1.693	1.219	1.184
MDC ICC(1,k)	5.276	4.694	3.378	3,.81
ICC(2,1)	0.864	0.887	0.936	0.935
ICC(2,1) 95% CI	[0.319, 0.952]	[0.571, 0.955]	[0.858, 0.967]	[0.896, 0.96]
SEM ICC(2,1)	2.548	2.298	1.69	1.646
MDC ICC(2,1)	7.063	6.368	4.684	4.563
ICC(2,k)	0.927	0.94	0.967	0.966
ICC(2,k) 95% CI	[0.461, 0.976]	[0.718, 0.977]	[0.923, 0.983]	[0.945, 0.98]
SEM ICC(2,k)	1.867	1.672	1.215	1.183
MDC ICC(2,k)	5.174	4.636	3.367	3.28
ICC(3,1)	0.93	0.93	0.948	0.936
ICC(3,1) 95% CI	[0.889, 0.957]	[0.888, 0.957]	[0.917, 0.968]	[0.897, 0.961]
SEM ICC(3,1)	1.821	1.807	1.513	1.632
MDC ICC(3,1)	5.048	5.009	4.195	4.525
ICC(3,k)	0.964	0.964	0.974	0.967
ICC(3,k) 95% CI	[0.941, 0.978]	[0.941, 0.978]	[0.957, 0.984]	[0.946, 0.98]
SEM ICC(3,k)	1.311	1.301	1.084	1.173
MDC ICC(3,k)	3.633	3.605	3.005	3.252

LoA = Limits of agreement, ICC = intraclass correlation coefficient, SEM = standard error of measurement, MDC = minimal detectable change

## Conclusion

For a comprehensive measurement evaluation, the systematic test–retest error (e.g., via mean bias line in BA analyses supplemented via paired-sample *t* test) as well as a random error analysis (e.g., via the MAE or MAPE_adj_ should supplement the reliability coefficient (e.g., ICC) quantification. Both, the systematic and random errors can be graphically illustrated via BA. Specifically, the difference between the 0-line and the actual average indicates the magnitude of systematic error , which is important to consider because a valid interpretation of ICCs requires the absence of systematic errors (Bartlett & Frost, 2008). Also, the MAE is confounded in the presence of a systematic error, as the random and systematic error messes up and might misleadingly indicate a large random error, as the MAE considers the absolute test–retest differences.

The additional presentation of LoAcc allows for an ad hoc content-related evaluation of both error sources and therefore solves open limitations in common BA analyses in which the LoAs all-time summarized the mean bias line (indicating a dependency). Furthermore, it facilitates the calculation and interpretation of further relevant reliability quantifications. However, to reach appropriate accuracy, the systematic bias should be viewed as an independent error source (also: see CTT), and both the systematic and the random error should be minimal. Only if both requirements are fulfilled independently, sufficient accuracy is indicated.

Measurement errors inherently occur in empirical data collections in a broad span of research topics. When seeking to ensure internal data validity, habituation effects must be controlled, and it is necessary to distinguish between systematic and random errors, which should be made transparent for reasonable data interpretation. The sole post-hoc and content-free agreement analysis of BA plots does not fulfill these requirements. By setting predefined acceptance criteria, researchers enhance the interpretability and applicability of BA analyses, making results more meaningful for scientific and practical use. To counteract this, this work introduced an easily applicable and generalized approach to define acceptable measurement error limits ad hoc. These allow for a detailed analysis of systematic and random errors in terms of test–retest reliability as a complement to commonly applied BA analyses.

Although the main objective here is to improve test–retest reliability reporting in which an agreement analysis is rather comparative than absolute, pre-set LoAccs can also be applied in validity or objectivity quantification, when having a gold standard measurement as a reference, or when comparing readings from different raters. The opportunity was previously outlined by Bland and Altman (1986) when introducing BA analyses and also apply to the introduced Shiny App. To allow for objectivity quantifications, the respective ICC(2,1 and 2,k) should be selected (Koo & Li, 2016; Shrout & Fleiss, 1979).

## Limitations

Although the software package automates the test–retest evaluation and allows pre-definition of acceptable measurement errors, one main concern that remains unsolved is the missing content-based classification of what can be considered acceptable. However, such acceptable error classification critically depends on the respective objectives of the study, assumed pre-post changes or effect sizes, and, for sure, the measurement. Since it was neither the objective of this study, nor might it be possible to provide guidance on acceptable limits in this work, the used 5% and 10% pre-set LoAccs should be considered as an example.

Nevertheless, using this software package allows for making transparent what authors assumed to be an acceptable test–retest measurement error as a complementation of already existing test–retest reliability quantifications, while future works should address how acceptable limits might be imputed from previous works, as it is done for, for instance, G-power sample size estimations, where previous effect sizes from related studies were used to calculated the required sample size. Another point of concern is that the application is limited to two columns. However, since this package was designed to improve the interpretability of BA analyses and allow for requested pre-set acceptable measurement error ranges, future work should address the query of implementing more than two data rows into an agreement analysis.

Lastly, in the CTT, error sources (T and E) were considered to be independent from each other. Although considering this clear separation as a necessary precondition for reliability and repeatability quantification might be disputable (see Generalizability theory), this discussion about the most appropriate test theory was not extensively discussed in this study, while both sources of error were quantified via the Shiny app independently.

## Supplementary Information

Below is the link to the electronic supplementary material.

Supplementary file1 (DOCX 29 KB)

## Data Availability

All data generated or analyzed during this study are included in this published article and its supplementary information files.

## Declarations

### Open practices statement and code availability

The code (and software) is accessible via the following link: https://cloud.uni-jena.de/s/Zf8XW62zQeyssEy as well as on the authors' Open Science Framework profile (osf.io/gbcea). The study was not preregistered.

## References

[CR1] Appelbaum, M., Cooper, H., Kline, R. B., Mayo-Wilson, E., Nezu, A. M., & Rao, S. M. (2018). Journal article reporting standards for quantitative research in psychology: The APA Publications and Communications Board task force report. *American Psychologist, 73*(1), 3–25. 10.1037/amp0000191 29345484

[CR2] Atkinson, G., & Nevill, A. M. (1998). Statistical methods for assessing measurement error (reliability) in variables relevant to sports medicine. *Sports Medicine, 26*(4), 217–238. 10.2165/00007256-199826040-00002 9820922

[CR3] Barnhart, H. X., Haber, M. J., & Lin, L. I. (2007). An overview on assessing agreement with continuous measurements. *Journal of Biopharmaceutical Statistics, 17*(4), 529–569. 10.1080/10543400701376480 17613641

[CR4] Bartlett, J. W., & Frost, C. (2008). Reliability, repeatability and reproducibility: analysis of measurement errors in continuous variables. *Ultrasound in Obstetrics & Gynecology, 31*(4), 466–475. 10.1002/uog.5256 18306169

[CR5] Bennett, R. J., Taljaard, D. S., Olaithe, M., Brennan-Jones, C., & Eikelboom, R. H. (2017). Evaluating random error in clinician-administered surveys: Theoretical considerations and clinical applications of interobserver reliability and agreement. *American Journal of Audiology, 26*(3), 191–201. 10.1044/2017_AJA-16-0100 28768319

[CR6] Bland, J. M., & Altman, D. G. (1995). Comparing methods of measurement: Why plotting difference against standard method is misleading. *The Lancet, 346*(8982), 1085–1087. 10.1016/S0140-6736(95)91748-9 7564793

[CR7] Bland, J. M., & Altman, D. G. (1997). Statistics notes: Cronbach’s alpha: Table 1. *BMJ, 314*(7080), 572. 10.1136/bmj.314.7080.572 PMC2126061 9055718

[CR8] Bland, J. M., & Altman, D. G. (1999). Measuring agreement in method comparison studies. *Statistical Methods in Medical Research, 8*(2), 135–160. 10.1177/096228029900800204 10501650

[CR9] Bland, J. M., & Altmann, D. G. (1986). Statistical methods of assessing agreement between two methods of clinical measurement. *Lancet, i*, 307–310. 2868172

[CR10] Bogdanov, M., Scott, J. N., Esfand, S. M., Boyle, B. W., Lees, T., Li, M., Woronko, S. E., Linton, S. R., Miller, C., Li, S., Bolton, P., Meisner, R. C., & Pizzagalli, D. A. (2025). Test-retest reliability and repeatability of behavioral and electrophysiological markers of cognitive control in an Eriksen Flanker Task. *Cognitive, Affective, & Behavioral Neuroscience, 25*(5), 1330–1358. 10.3758/s13415-025-01336-7 40790001

[CR11] Brandl, A., Acikalin, E., Bartsch, K., Wilke, J., & Schleip, R. (2024a). Reliability and validity of an app-assisted tissue compliance meter in measuring tissue stiffness on a phantom model. *PeerJ, 12*, e17122. 10.7717/peerj.17122 PMC10924762 38464760

[CR12] Brandl, A., Wilke, J., Horstmann, T., Reer, R., Egner, C., Schmidt, T., & Schleip, R. (2024b). Quantifying thoracolumbar fascia deformation to discriminate acute low back pain patients and healthy individuals using ultrasound. *Scientific Reports, 14*(1), 20044. 10.1038/s41598-024-70982-7 PMC11362488 39209953

[CR13] Brennan, R. L. (2010). Generalizability theory and classical test theory. *Applied Measurement in Education, 24*(1), 1–21. 10.1080/08957347.2011.532417

[CR14] Cabarkapa, D., Smith, R., Chowning, L., Neltner, T., Johnson, Q. R., Yang, Y., & Munce, T. A. (2025). Reliability of vertical jump force-time metrics in collegiate athletes compared to recreationally active individuals. *Life, 15*(12), 1830. 10.3390/life15121830 PMC12733763 41465769

[CR15] Cataldi, D., Bennett, J. P., Quon, B. K., Liu, Y. E., Heymsfield, S. B., Kelly, T., & Shepherd, J. A. (2022). Agreement and precision of deuterium dilution for total body water and multicompartment body composition assessment in collegiate athletes. *Journal of Nutrition, 152*(9), 2048–2059. 10.1093/jn/nxac116 35665820

[CR16] Cesar, G. M., Buster, T. W., & Burnfield, J. M. (2021). Test-retest reliability and minimal detectable change of the computerized dynamic posturography PROPRIO for adults with chronic traumatic brain injury. *Disability and Rehabilitation, 43*(14), 2038–2044. 10.1080/09638288.2019.1688872 31724889

[CR17] Collie, A., Maruff, P., Darby, D. G., & McStephen, M. (2003). The effects of practice on the cognitive test performance of neurologically normal individuals assessed at brief test–retest intervals. *Journal of the International Neuropsychological Society, 9*(3), 419–428. 10.1017/S1355617703930074 12666766

[CR18] Collins, L. M. (2007). Research design and methods. In *Encyclopedia of gerontology* (pp. 433–442). Elsevier. 10.1016/B0-12-370870-2/00162-1

[CR19] Cronbach, L. J. (1947). Test “Reliability”: Its meaning and determination. *Psychometrika, 12*(1), 1–16. 10.1007/BF02289289 20293842

[CR20] Cronbach, L. J. (1951). Coefficient alpha and the internal structure of tests. *Psychometrika, 16*(3), 297–334. 10.1007/BF02310555

[CR21] Cronbach, L. J., Gleser, G. C., Nanda, H., & Rajaratnam, N. (1972). *the dependability of behavioral measurements: Theory of generalizability for scores and profiles*. Wiley.

[CR22] Currell, K., & Jeukendrup, A. E. (2008). Validity, reliability and sensitivity of measures of sporting performance. *Sports Medicine, 38*(4), 297–316. 10.2165/00007256-200838040-00003 18348590

[CR23] Del Rossi, G., Malaguti, A., & Del Rossi, S. (2014). Practice effects associated with repeated assessment of a clinical test of reaction time. *Journal of Athletic Training, 49*(3), 356–359. 10.4085/1062-6059-49.2.04 PMC4080596 24673236

[CR24] Dikmen, S. S., Heaton, R. K., Grant, I., & Temkin, N. R. (1999). Test-retest reliability and practice effects of expanded Halstead-Reitan neuropsychological test battery. *Journal of the International Neuropsychological Society : JINS, 5*(4), 346–356. 10349297

[CR25] Doğan, N. Ö. (2018). *Bland-Altman analysis: A paradigm to understand correlation and agreement.* 10.1016/j.tjem.2018.09.001 PMC6261099 30533555

[CR26] Donath, L., Ludyga, S., Hammes, D., Rossmeissl, A., Andergassen, N., Zahner, L., & Faude, O. (2017). Absolute and relative reliability of acute effects of aerobic exercise on executive function in seniors. *BMC Geriatrics, 17*(1), 247. 10.1186/s12877-017-0634-x PMC5657086 29070027

[CR27] Eileen De Monte, V., Malke Geffen, G., & Kwapil, K. (2005). Test-retest reliability and practice effects of a rapid screen of mild traumatic brain injury. *Journal of Clinical and Experimental Neuropsychology, 27*(5), 624–632. 10.1080/13803390490918589 16019639

[CR28] Estevis, E., Basso, M. R., & Combs, D. (2012). Effects of practice on the Wechsler adult intelligence scale-IV across 3- and 6-month intervals. *The Clinical Neuropsychologist, 26*(2), 239–254. 10.1080/13854046.2012.659219 22353021

[CR29] Food and Drug Administration (FDA). (2001). Guidance for industry: Bioanalytical method validation. http://Ww.Fda.Gov/Cder/Guidance/Index.Htm

[CR30] Galpin, A. J., Li, Y., Lohnes, C. A., & Schilling, B. K. (2008). A 4-week choice foot speed and choice reaction training program improves agility in previously non-agility trained, but active men and women. *Journal of Strength and Conditioning Research, 22*(6), 1901–1907. 10.1519/JSC.0b013e3181887e3f 18978618

[CR31] Gamer, M., & Lemon, J. (2019). Various coefficients of interrater reliability and agreement. 10.32614/CRAN.package.irr

[CR32] Giavarina, D. (2015). Understanding Bland altman analysis. *Biochemia Medica, 25*(2), 141–151. 10.11613/BM.2015.015 PMC4470095 26110027

[CR33] Gibbon, J. R., Parry, S. W., Witham, M. D., Yarnall, A., & Frith, J. (2022). Feasibility, reliability and safety of self-assessed orthostatic blood pressure at home. *Age and Ageing, 51*(7). 10.1093/ageing/afac153 35776671

[CR34] Grgic, J., Lazinica, B., Schoenfeld, B. J., & Pedisic, Z. (2020). Test–retest reliability of the one-repetition maximum (1RM) strength assessment: A systematic review. *Sports Medicine - Open, 6*(1), 31. 10.1186/s40798-020-00260-z PMC7367986 32681399

[CR35] Haghayegh, S., Kang, H.-A., Khoshnevis, S., Smolensky, M. H., & Diller, K. R. (2020). A comprehensive guideline for Bland–Altman and intra class correlation calculations to properly compare two methods of measurement and interpret findings. *Physiological Measurement, 41*(5), 055012. 10.1088/1361-6579/ab86d6 32252039

[CR36] Harvill, L. M. (1991). Standard error of measurement. *Educational Measurement: Issues and Practice, 10*(2), 33–41. 10.1111/j.1745-3992.1991.tb00195.x

[CR37] Hopkins, W. G. (2000). Measures of reliability in sports medicine and science. *Sports Medicine, 30*(1), 1–15. 10.2165/00007256-200030010-00001 10907753

[CR38] Hwang, K., De Silva, A., Simpson, J. A., LoGiudice, D., Engel, L., Gilbert, A. S., Croy, S., & Haralambous, B. (2022). Video-interpreting for cognitive assessments: An intervention study and micro-costing analysis. *Journal of Telemedicine and Telecare, 28*(1), 58–67. 10.1177/1357633X20914445 32228142

[CR39] International Organization of Standardization. (2023). ISO 5725-1:2023(en) accuracy (trueness and precision) of measurement methods and results - Part 1: General principles and definitions. https://Www.Iso.Org/Obp/Ui/#iso:Std:Iso:5725:-1:Ed-2:V1:En

[CR40] Jogie, P., Rahja, M., van den Berg, M., Salway, J., & Laver, K. (2025). Evaluating goal attainment scaling for individuals with mild cognitive impairment or dementia in rehabilitation settings. *Archives of Rehabilitation Research and Clinical Translation, 7*(4), 100501. 10.1016/j.arrct.2025.100501 PMC12750371 41477101

[CR41] Koo, T. K., & Li, M. Y. (2016). A guideline of selecting and reporting intraclass correlation coefficients for reliability research. *Journal of Chiropractic Medicine, 15*(2), 155–163. 10.1016/j.jcm.2016.02.012 PMC4913118 27330520

[CR42] Kottner, J., & Streiner, D. L. (2011). The difference between reliability and agreement. *Journal of Clinical Epidemiology, 64*(6), 701–702. 10.1016/j.jclinepi.2010.12.001 21411278

[CR43] Krabbe, P. F. M. (2017). Classical test theory. In *The measurement of health and health status* (pp. 153–170). Elsevier. 10.1016/B978-0-12-801504-9.00009-X

[CR44] Lachin, J. M. (2004). The role of measurement reliability in clinical trials. *Clinical Trials, 1*(6), 553–566. 10.1191/1740774504cn057oa 16279296

[CR45] Lamb, K. (1998). Test-retest reliability in quantitative physical education research: A commentary. *European Physical Education Review, 4*(2), 145–152.

[CR46] Lempke, L. B., Shumski, E. J., Prato, T. A., & Lynall, R. C. (2023). Reliability and minimal detectable change of the standardized assessment of reaction time. *Journal of Athletic Training, 58*(6), 579–587. 10.4085/1062-6050-0391.22 PMC10496444 36252207

[CR47] Lin, L. I.-K. (1989). A concordance correlation coefficient to evaluate reproducibility. *Biometrics, 45*(1), 255–268 https://www.jstor.org/stable/2532051 2720055

[CR48] Lo, A. H. Y., Humphreys, M., Byrne, G. J., & Pachana, N. A. (2012). Test–retest reliability and practice effects of the Wechsler Memory Scale-III. *Journal of Neuropsychology, 6*(2), 212–231. 10.1111/j.1748-6653.2011.02023.x 22257421

[CR49] Lord, F. M., & Novick, M. R. (1986). *Statistical theories of mental test scores*. Addison-Wesley.

[CR50] Luque Laguna, P. A., Combes, A. J. E., Streffer, J., Einstein, S., Timmers, M., Williams, S. C. R., & Dell’Acqua, F. (2020). Reproducibility, reliability and variability of FA and MD in the older healthy population: A test-retest multiparametric analysis. *NeuroImage: Clinical, 26*, 102168. 10.1016/j.nicl.2020.102168 PMC7011084 32035272

[CR51] Lynall, R. C., Johnson, R. S., Lempke, L. B., & Schmidt, J. D. (2021). Test–retest reliability of a functional reaction time assessment battery. *Journal of Sport Rehabilitation, 30*(8), 1237–1241. 10.1123/jsr.2021-0021 33952713

[CR52] Marenco, S., Rawlings, R., Rohde, G. K., Barnett, A. S., Honea, R. A., Pierpaoli, C., & Weinberger, D. R. (2006). Regional distribution of measurement error in diffusion tensor imaging. *Psychiatry Research: Neuroimaging, 147*(1), 69–78. 10.1016/j.pscychresns.2006.01.008 PMC1941705 16797169

[CR53] Medvedeva, A., Galli, G., Petrovskaya, A., & Medvedev, O. N. (2023). A novel application of generalizability theory to evaluate the reliability of the recognition memory test. *Current Psychology, 42*(20), 17020–17032. 10.1007/s12144-022-02886-6

[CR54] Miller, M. D. (2009). Classical test theory reliability. In *International encyclopedia of education* (3rd ed., pp. 27–30). 10.1016/B978-0-08-044894-7.00235-9

[CR55] Nevill, A. M., & Atkinson, G. (1997). Assessing agreement between measurements recorded on a ratio scale in sports medicine and sports science. *British Journal of Sports Medicine, 31*(4), 314–318. 10.1136/bjsm.31.4.314 PMC1332566 9429009

[CR56] Palsma, T., Claassen, J. A. H. R., Richard, E., & De Heus, R. A. A. (2025). Day-to-day blood pressure variability in older persons – Optimizing measurement. *Journal of Hypertension, 43*(6), 970–975. 10.1097/HJH.0000000000003975 PMC12052059 40008517

[CR57] Palta, M., Chen, H.-Y., Kaplan, R. M., Feeny, D., Cherepanov, D., & Fryback, D. G. (2011). Standard error of measurement of 5 health utility indexes across the range of health for use in estimating reliability and responsiveness. *Medical Decision Making, 31*(2), 260–269. 10.1177/0272989X10380925 PMC3607511 20935280

[CR58] Patino, C. M., & Ferreira, J. C. (2018). Internal and external validity: Can you apply research study results to your patients? *Jornal Brasileiro de Pneumologia, 44*(3), 183–183. 10.1590/s1806-37562018000000164 PMC6188693 30043882

[CR59] Piscicelli, C., Barra, J., Davoine, P., Chrispin, A., Nadeau, S., & Pérennou, D. (2015). Inter- and intra-rater reliability of the visual vertical in subacute stroke. *Stroke, 46*(7), 1979–1983. 10.1161/STROKEAHA.115.009610 26022631

[CR60] Ramos-Loyo, J., González-Garrido, A. A., Llamas-Alonso, L. A., & Sequeira, H. (2022). Sex differences in cognitive processing: An integrative review of electrophysiological findings. *Biological Psychology, 172*, 108370. 10.1016/j.biopsycho.2022.108370 35662578

[CR61] Reimers, S., & Maylor, E. A. (2006). Gender effects on reaction time variability and trial-to-trial performance: Reply to Deary and Der (2005). *Aging, Neuropsychology, and Cognition, 13*(3–4), 479–489. 10.1080/138255890969375 16887784

[CR62] Sanders, L. M. J., Hortobágyi, T., Balasingham, M., Van der Zee, E. A., & van Heuvelen, M. J. G. (2018). Psychometric properties of a flanker task in a sample of patients with dementia: A pilot study. *Dementia and Geriatric Cognitive Disorders Extra, 8*(3), 382–392. 10.1159/000493750 PMC6243949 30483304

[CR63] Schworer, E. K., Soltani, A., Altaye, M., Fidler, D. J., & Esbensen, A. J. (2023). Cognitive flexibility assessment in youth with down syndrome: Reliability, practice effects, and validity. *Research in Developmental Disabilities, 133*, 104416. 10.1016/j.ridd.2022.104416 PMC9852016 36603310

[CR64] Seamon, B. A., Kautz, S. A., Bowden, M. G., & Velozo, C. A. (2022). Revisiting the concept of minimal detectable change for patient-reported outcome measures. *Physical Therapy, 102*(8). 10.1093/ptj/pzac068 PMC9361333 35670017

[CR65] Shrout, P. E., & Fleiss, J. L. (1979). Intraclass correlations: Uses in assessing rater reliability. *Psychological Bulletin, 86*(2), 420–428. 10.1037/0033-2909.86.2.420 18839484

[CR66] Taule, T., Eide, I. S., Fjær, L., Myrberget, M.-A., Oseland, M. S., Renså, M. A., Revheim, T., Tysnes, O.-B., Aßmus, J., & Rekand, T. (2023). Norwegian version of the Edinburgh cognitive and behavioural ALS screen: Construct validity, internal consistency, inter-rater, and test-retest reliability. *PLOS ONE, 18*(5), e0285307. 10.1371/journal.pone.0285307 PMC10159149 37141321

[CR67] Theisen, M. E., Rapport, L. J., Axelrod, B. N., & Brines, D. B. (1998). Effects of practice in repeated administrations of the Wechsler memory scale-revised in normal adults. *Assessment, 5*(1), 85–92. 10.1177/107319119800500110 9458344

[CR68] Thompson, B. (1992). Two and one-half decades of leadership in measurement and evaluation. *Journal of Counseling & Development, 70*(3), 434–438. 10.1002/j.1556-6676.1992.tb01631.x

[CR69] Vetter, T. R., & Schober, P. (2018). Agreement analysis: What he said, she said versus you said. *Anesthesia & Analgesia, 126*(6), 2123–2128. 10.1213/ANE.0000000000002924 29677066

[CR70] Warneke, K., Gronwald, T., Wallot, S., Magno, A., Hillebrecht, M., & Wirth, K. (2025a). discussion on the validity of commonly used reliability indices in sports medicine and exercise science - A critical review with data simulations. *European Journal of Applied Physiology.* 10.1007/s00421-025-05720-6 PMC12174282 39939564

[CR71] Warneke, K., Lebelt, N., Wallot, S., Lohmann, L. H., Afonso, J., & Herbsleb, M. (2025b). (Mis-)understanding external validity – A narrative literature review with guideline synthesis on how to report reliability in sport and health science. *Football Studies, 100011*. 10.1016/j.footst.2025.100011

[CR72] Warneke, K., Oraze, M., Herbsleb, M., Afonso, J., & Wallot, S. (2025c). Do Sex differences influence test habituation and internal data validity in neurocognitive testing? A blinded measurement error analysis*. Neuroscience, 593*, 106–121. 10.1016/j.neuroscience.2025.12.007 41365460

[CR73] Warneke, K., Oraze, M., Plöschberger, G., Herbsleb, M., Afonso, J., & Wallot, S. (2025d). When testing becomes learning - Underscoring the relevance of habituation to improve internal validity of common neurocognitive tests. *European Journal of Neuroscience, 61*(8), e70117. 10.1111/ejn.70117 PMC12022537 40275720

[CR74] Warneke, K., Siegel, S. D., Afonso, J., & Wallot, S. (2025e). What the mean absolute percentage error (MAPE) should adopt from Bland–Altman analyses. *German Journal of Exercise and Sport Research.* 10.1007/s12662-025-01084-3

[CR75] Weir, J. P. (2005). Quantifying test-retest reliability using the intraclass correlation coefficient and the SEM. *The Journal of Strength and Conditioning Research, 19*(1), 231. 10.1519/15184.1 15705040

[CR76] Westheim, F., Gløersen, Ø., Harper, D., Laugsand, H., & Eriksrud, O. (2023). Reliability of phase-specific outcome measurements in change-of-direction tests using a motorized resistance device. *Frontiers in Sports and Active Living, 5*, 1212414. 10.3389/fspor.2023.1212414 PMC10626501 37936876

[CR77] White, N., Flannery, L., McClintock, A., & Machado, L. (2019). Repeated computerized cognitive testing: Performance shifts and test–retest reliability in healthy older adults. *Journal of Clinical and Experimental Neuropsychology, 41*(2), 179–191. 10.1080/13803395.2018.1526888 30320531

[CR78] Wilke, J., & Vogel, O. (2020). Computerized cognitive training with minimal motor component improves lower limb choice-reaction time. *Journal of Sports Science & Medicine, 19*(3), 529–534. PMC7429437 32874106

[CR79] Wilke, J., Vogel, O., & Ungricht, S. (2020). Can we measure perceptual-cognitive function during athletic movement? A framework for and reliability of a sports-related testing battery. *Physical Therapy in Sport, 43*, 120–126. 10.1016/j.ptsp.2020.02.016 32145687

[CR80] Willmott, C., & Matsuura, K. (2005). Advantages of the mean absolute error (MAE) over the root mean square error (RMSE) in assessing average model performance. *Climate Research, 30*, 79–82. 10.3354/cr030079

[CR81] Wright, E. M., & Royston, P. (1999). Calculating reference intervals for laboratory measurements. *Statistical Methods in Medical Research, 8*(2), 93–112. 10.1177/096228029900800202 10501648

