# Supplementary material for: How much habituation is enough? An R application for ad hoc content-related limits of acceptance in behavioral research
Source: Behav Res Methods. 2026 Sep 30;58(11):307. doi: 10.3758/s13428-026-03170-2 (PMC13627198; doi:10.3758/s13428-026-03170-2)
Supplement: Supplementary file 1 [file 13428_2026_3170_MOESM1_ESM.docx]

**SUPPLEMENTAL MATERIAL**

**Table S1.** simulated data to illustrate the different scenarios of the Bland-Altman Analysis provided in the manuscript.

| **ID** | **High_Sys/**  **Low_Rand1** | **High_Sys/**  **Low_Rand2** | **Low_Sys/**  **High_Rand1** | **Low_Sys/**  **High_Rand2** | **High_Sys/**  **High_Rand1** | **High_Sys/**  **High_Rand2** | **Low_Sys/**  **Low_Rand1** | **Low_Sys/**  **Low_Rand2** |
| --- | --- | --- | --- | --- | --- | --- | --- | --- |
| 1 | 22.8 | 25.7 | 18.3 | 16.3 | 26 | 28.1 | 29.1 | 28 |
| 2 | 24.1 | 28.5 | 28.4 | 29.1 | 24.9 | 29.6 | 23.9 | 24.2 |
| 3 | 31.2 | 34.9 | 25.6 | 25.8 | 24.8 | 29.1 | 20.1 | 19.7 |
| 4 | 25.3 | 28.8 | 20.4 | 17.6 | 30.5 | 31.6 | 25.7 | 25.2 |
| 5 | 25.5 | 28.1 | 30 | 29.8 | 24.1 | 27.9 | 24.4 | 23 |
| 6 | 31.9 | 35.8 | 26.7 | 31 | 31.1 | 39.4 | 25 | 25 |
| 7 | 26.8 | 29.7 | 23.8 | 25.2 | 18.8 | 24.2 | 26.5 | 25.4 |
| 8 | 19.9 | 21.4 | 28.6 | 28.7 | 27.3 | 31.5 | 23.5 | 21 |
| 9 | 22.3 | 25.7 | 28.5 | 27.2 | 25.5 | 28.2 | 27.6 | 27 |
| 10 | 23.2 | 28.6 | 28.3 | 22.1 | 25.9 | 23.7 | 24.1 | 25.5 |
| 11 | 29.9 | 33 | 27.8 | 31.1 | 26.5 | 33.9 | 26.3 | 25.5 |
| 12 | 26.4 | 31.4 | 27.2 | 22.8 | 23 | 22.6 | 29.4 | 30.3 |
| 13 | 26.6 | 28.2 | 24.8 | 27 | 23.7 | 29.9 | 26.7 | 24.3 |
| 14 | 25.4 | 29.4 | 23.8 | 29.5 | 20.9 | 30.7 | 23.7 | 23.6 |
| 15 | 22.8 | 27.6 | 23.5 | 19.1 | 20.7 | 20.4 | 29.6 | 30.4 |
| 16 | 32.1 | 36.6 | 22.2 | 24.3 | 26.2 | 32.3 | 29 | 29.4 |
| 17 | 27 | 31.1 | 24.2 | 23.4 | 26.8 | 30 | 27.2 | 27.4 |
| 18 | 17.1 | 20.2 | 19.9 | 15.2 | 25.2 | 24.5 | 26 | 25 |
| 19 | 27.8 | 30.5 | 33.7 | 29.1 | 28.7 | 28.1 | 22.5 | 21.2 |
| 20 | 23.1 | 25.6 | 29.8 | 25 | 33.2 | 32.4 | 30.4 | 28.9 |
| 21 | 20.7 | 24.9 | 20.5 | 18.9 | 23 | 25.4 | 22.6 | 22.8 |
| 22 | 24.1 | 26.7 | 23.4 | 19 | 15.8 | 15.4 | 33.7 | 32.3 |
| 23 | 20.9 | 24.2 | 23.1 | 25.2 | 29 | 35.1 | 31.1 | 30.4 |
| 24 | 22.1 | 25.7 | 28.1 | 34.4 | 22.2 | 32.5 | 24.1 | 23.7 |
| 25 | 22.5 | 29.3 | 24.7 | 20.8 | 22.2 | 22.4 | 20.9 | 23.7 |

High_Sys = high systematic bias. Low_Rand = low random error. 1 = day 1. 2 = day 2. ID = participant identification number

**Table S2.** Reliability metrics for the different scenarios calculated on the data from Table S2.

| **Pair** | **Mean.SD1** | **Mean.SD2** | **ICC.Agreement**  **ICC2** | **ICC.Consistency**  **ICC3** | **SEM**  **Agreement** | **MDC**  **Agreement** | **SEM**  **Consistency** | **MDC**  **Consistency** | **MAE** | **MAPE** | **LoA** | **Bias.p.value** |
| --- | --- | --- | --- | --- | --- | --- | --- | --- | --- | --- | --- | --- |
| **High_Sys_Low_Rand1 vs High_Sys_Low_Rand2** | 24.86 ± 3.79 | 28.46 ± 4.03 | 0.606 | 0.613 | 2.378 | 6.59 | 2.354 | 6.526 | 3.604 | 13.65 | 1.297 to 5.911 | 0 |
| **Low_Sys_High_Rand1 vs Low_Sys_High_Rand2** | 25.41 ± 3.68 | 24.7 ± 5.12 | 0.693 | 0.685 | 2.038 | 5.649 | 2.064 | 5.723 | 2.98 | 12.24 | -7.637 to 6.221 | 0.32667 |
| **High_Sys_High_Rand1 vs High_Sys_High_Rand2** | 25.04 ± 3.89 | 28.36 ± 5.18 | 0.512 | 0.511 | 2.718 | 7.534 | 2.719 | 7.537 | 3.748 | 13.58 | -3.628 to 10.26 | 0.00009 |
| **Low_Sys_Low_Rand1 vs Low_Sys_Low_Rand2** | 26.12 ± 3.31 | 25.72 ± 3.26 | 0.934 | 0.932 | 0.853 | 2.365 | 0.866 | 2.4 | 0.968 | 3.82 | -2.699 to 1.883 | 0.09367 |

High_Sys = high systematic bias. Low_Rand = low random error. 1 = day 1. 2 = day 2. ID = participant identification number. SD = standard deviation. ICC = intraclass correlation coefficient. SEM = standard error of measurement. MDC = minimal detectable change. MAE = mean absolute error. MAPE = mean absolute percentage error. LoA = Limits of Agreement. Bias.p.value = p value for systematic bias evaluation
